# Genome‐wide identification of olfactory receptor and odorant‐binding protein gene families and their roles in Heliothine chemosensory evolution

**DOI:** 10.1111/imb.70052

**Published:** 2026-06-28

**Authors:** Rong Guo, Boyang Ni, Megan L. Fritz

**Affiliations:** ^1^ Department of Entomology University of Maryland College Park Maryland USA; ^2^ Computational Biology, Bioinformatics and Genomics Program, Department of Biological Sciences University of Maryland College Park Maryland USA; ^3^ Institute for Quantitative Biology, Biochemistry and Biotechnology University of Edinburgh Edinburgh UK

**Keywords:** Heliothinae, odorant‐binding proteins (OBPs), odorant receptors (ORs)

## Abstract

Chemosensory systems play key roles in the survival and reproductive success of insects. Two large and diverse chemosensory gene families, odorant receptors (ORs) and odorant‐binding proteins (OBPs), play critical roles in insect chemosensation and mediate odour‐guided behaviours. In the process of insect chemosensation, odorants from the environment pass through pores in the antennal sensilla and become soluble in the sensillar lymph, either directly on contact or by binding to an OBP. Solubilized odour molecules diffuse through the lymph until they reach and activate their cognate ORs, sending electrophysiological signals to the insect brain. To better understand the evolutionary roles of OR and OBP gene families among members of the Heliothinae, we systematically characterized these two gene families in *Chloridea virescens* (Lepidoptera: Noctuidae). A total of 81 ORs and 49 OBPs were identified genome‐wide. Based on the number and positions of conserved cysteine residues, the OBPs were classified into three types: 34 Classic OBPs, 8 Minus‐C OBPs and 7 Plus‐C OBPs. Phylogenetic analyses identified potential gene duplications and losses within OR and OBP gene families among members of the Heliothinae, which may be associated with differences in their volatile sensation and olfactory behaviours. Further motif and structural analyses identified a conserved region that was unique among pheromone receptors and predicted as key residues of the binding pocket, implying its critical role in pheromone detection. Future work should focus on experimentally validating its function. Overall, our findings provide important insights into how chemosensory gene evolution contributes to ecological adaptation and reproductive isolation in the Heliothine moths.

## Graphical abstract

- Genome‐wide systematic annotation identified 81 odorant receptors (ORs) and 49 odorant‐binding proteins (OBPs) in the tobacco budworm, *Chloridea virescens*.
- Phylogenetic analyses revealed chemosensory gene diversification in Heliothinae and extensive lineage‐specific duplications in *C. virescens*, consistent with its adaptation to diverse host plant chemical signals.
- Structural and motif analyses identified a conserved motif within the binding pocket of pheromone receptors, highlighting its critical role in ligand binding and pheromone specificity.

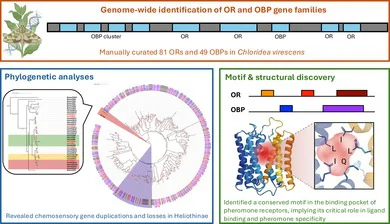

## INTRODUCTION

The chemosensory system plays a crucial role in many insect behaviours, such as feeding, mating, finding oviposition sites and avoiding harmful factors such as predators (Leal, 2013). Accurate sensation and recognition of odorants are critical to fitness, particularly for pheromone components and host plant odorants (Pelosi et al., 2018; Zhang, Walker, & Wang, 2015). Generally, odorants from the environment are detected by olfactory sensory neurons (OSNs) housed in multiporous cuticular hairs on insect antennae. Hydrophobic odorants are first adsorbed to the waxy cuticular surface and then pass through pores of the olfactory sensillum. The molecules become water‐soluble through binding to odorant‐binding proteins (OBPs) in the lymph fluid, and they are carried to OSN dendrites, finally binding with odorant receptors (ORs) on the dendritic membrane (Sakurai et al., 2014). The binding triggers the activation of ion channels and converts chemical signals into electrical signals.

Insect ORs have seven transmembrane domains but a topology that is distinct from mammalian ORs, which are considered to be G protein‐coupled receptors (GPCRs) (Carraher et al., 2015; Zhang et al., 2025). Membrane topology analysis of the insect ORs indicates that they have an intracellular N‐terminus and an extracellular C‐terminus, while other GPCRs have the C‐termini inside the cell (Carraher et al., 2015; Smart et al., 2008; Tsitoura et al., 2010). Insect ORs function as a hetero‐tetramer in OSNs: a co‐receptor Orco, previously known as OR83b (Vosshall & Hansson, 2011), which is highly conserved between species (Krieger et al., 2003), and an odorant‐binding subunit (i.e., a tuning OR). The numbers of odorant‐binding subunits vary widely between species. For example, head lice contain as few as 10 OR genes while ants contain as many as 300 OR genes (Kirkness et al., 2010; Smith et al., 2011).

Pheromone receptors (PRs) are highly specialized ORs that are involved in pheromone‐based sexual communications, and the molecular mechanisms of pheromone recognition have been characterized in several lepidopteran families, such as Bombycidae (Sakurai et al., 2014), Crambidae (Wanner et al., 2010) and Noctuidae (Jiang et al., 2014; Liu et al., 2013). Female pheromones are a complex blend of volatiles, where the exact combinations of compounds and their ratios make up a species‐specific blend, to which the conspecific male's PRs respond. The species specificity of ligand‐receptor recognition enables precise mating and maintains reproductive isolation and species boundaries (Roelofs & Comeau, 1969), so even modest changes in PRs can lead to shifts in mating preferences or speciation (Cao et al., 2021).

OBPs are small (ca. 150 amino acids), water‐soluble proteins secreted into the lymph fluid surrounding the OSNs. The first OBPs were identified as pheromone‐binding proteins (PBPs) in moths (Vogt & Riddiford, 1981). They are widely believed to bind with different odorants and solubilize and transport them across the lymph fluid to ORs expressed on the dendritic membrane (Pelosi & Maida, 1995; Vogt & Riddiford, 1981). In addition to this major function, OBPs have been proposed to protect odorants from degradation or deactivation by odorant‐degrading enzymes (Leal, 2013). A study by Larter et al. (2016) also revealed a novel role for OBPs in buffering sudden changes in the odorant levels of the environment, suggesting that OBPs have a broader functional role than odorant transport alone. Generally, OBPs can be classified into different sub‐families according to their putative functions: PBP, having the ability to bind to pheromone components (Vogt & Riddiford, 1981); general odorant‐binding protein (GOBP) that were considered to respond to general odorants, especially volatiles from host plants (Khuhro et al., 2017); and antennal binding protein (ABP; Gong et al., 2009). The 3D structural features of insect OBPs have attracted interest as a potential target for pest control strategies (Venthur & Zhou, 2018; Zhou et al., 2010). Structurally, insect OBPs can also be categorized into classes (‘Classic’, ‘C‐minus’, ‘C‐plus’ and ‘Duplex or Dimer’) based on the number and pattern of cysteines. The six conserved cysteines of Classic OBPs form three disulphide bonds that stabilize their tertiary structure and help define a hydrophobic binding cavity (Gong et al., 2009; Hekmat‐Scafe et al., 2002; Manoharan et al., 2013; Vieira et al., 2007; Vieira & Rozas, 2011; Zhou et al., 2004).

Heliothine moths (Lepidoptera: *Heliothinae*) include some of the world's most devastating pest species. *Chloridea virescens* (previously *Heliothis virescens*), also known as tobacco budworm, is an important agricultural pest of many crops in the United States, attacking cotton, flax, soybean and tobacco in its larval stages (Blanco et al., 2007; Cunningham & Zalucki, 2014; Kogan et al., 1989). Two other closely related Heliothine species, *Helicoverpa zea* (Lepidoptera: Noctuidae) and *Helicoverpa armigera* (Lepidoptera: Noctuidae), are also significant agricultural pests in different regions of the world: *H. zea* in the Americas, while *H. armigera* in temperate Asia, Africa, South America and Australia (Pearce et al., 2017; Sharma, 2005). Although *C. virescens*, *H. zea* and *H. armigera* are all polyphagous, with overlapping host plant use, *C. virescens* (tobacco budworm) shows a preference for laying eggs on Solanaceae (e.g., tobacco and tomato), while the other two *Helicoverpa* do not (Cunningham & Zalucki, 2014). As their chemosensory systems play a critical role in mate finding and host plant recognition, a better understanding of the chemosensory receptors could contribute to studying the evolutionary process of speciation and exploring environmentally friendly approaches for pest control.

The insect chemosensory gene repertoire evolves through an extremely rapid birth‐and‐death process, with expansions driven by gene duplication and neofunctionalization, and contractions caused by pseudogenization and deletion (Sánchez‐Gracia et al., 2009). Comparative genomic studies have shown that insect chemosensory genes appear in tandem arrays and likely originate by unequal crossing over, then evolve independently from each other (Eyun et al., 2017; Nozawa & Nei, 2007; Sánchez‐Gracia et al., 2009; Vieira et al., 2007). To study the evolutionary divergence of OR and OBP gene families among members of the Heliothinae, we manually curated OR and OBP genes in the publicly available reference genome of *C. virescens* (GenBank assembly accession: GCA_002382865.1; Fritz et al., 2018). The number, positions and sequences of ORs and OBPs were identified. OBP genes were then classified into three different types (Classic, Minus‐C and Plus‐C), according to the number and position of conserved cysteine residues. Phylogenetic relationships among chemosensory genes from the Heliothinae were examined to identify gene duplication, loss and sequence evolution and infer the gene family evolutionary histories. Additional structural analysis revealed that sequence motif may be structurally or functionally important to sensation and detection of odorants critical to maintenance of Heliothine species boundaries.

## EXPERIMENTAL PROCEDURES

### Genome‐wide identification of OR and OBP genes

The webapollo platform hosted by the insect 5000 genomes initiative (i5k; Poelchau et al., 2014) was used for manual curation of OR and OBP genes in the reference genome of *C. virescens* (GenBank assembly accession: GCA_002382865.1; Fritz et al., 2018). Publicly available OR and OBP proteins from closely related species, *H. zea*, *H. armigera* and *Bombyx mori* (Lepidoptera: Bombycidae), and some chemosensory receptor proteins of *C. virescens* (in NCBI) were used to identify specific protein motifs or BLAST against the genome assemblies. Existing gene models from a structural annotation were either accepted or modified in light of both these tblastn results and guidance from spliced RNA‐seq reads from adult male and female antennae (BioProject accession number: PRJNA49697; USDA‐ARS‐BCIRL & USDA‐ARS‐SIMRU, 2010; GenBank accession numbers: SRR6417884 and SRR6417880). The genes were named according to the best‐‐matched ORs/OBPs of *H. zea* and *H. armigera*, which were identified and manually curated by Pearce et al. (2017). The predicted *C. virescens* OBP protein sequences were aligned to one another with ClustalW (Thompson et al., 2003) and were classified as ‘Classic’, ‘Minus‐C’ and ‘Plus‐C’ OBPs according to the conserved number and spacing of cysteine residues in the peptide sequences (Gong et al., 2009; Manoharan et al., 2013; Vieira & Rozas, 2011).

### Sequence alignment and phylogenetic tree construction

To study the evolutionary divergence of OR and OBP gene families among members of the Heliothinae, we compared the OR and OBP protein sequences from *C. virescens* to closely related Heliothine species: *Chloridea subflexa* (previously *Heliothis subflexa*) (Lepidoptera: Noctuidae), *H. zea*, *H. armigera* and *Helicoverpa assulta* (Lepidoptera: Noctuidae). As a lepidopteran species with one of the best‐characterized genomes (International Silkworm Genome Consortium, 2008), *B. mori* was chosen as an outgroup to identify lineage‐specific gene duplications, losses or functional innovations. In Pearce et al. (2017), 83 ORs, one Orco and 40 OBPs, and 81 ORs, one Orco, and 40 OBPs had been manually annotated in the reference genomes of *H. armigera* and *H. zea* (GenBank assembly accessions: GCA_002156985.1, GCA_002150865.1), respectively. For the phylogenetic analysis, four and eight ORs of *H. armigera* and *H. zea*, respectively, were removed as the protein translations did not start with methionine (M). In addition to the 80 annotated *C. virescens* ORs, the protein sequence of HvirOR11 (GenBank accession number: QLF97429.1), which could not be found in the assembly due to its quality, was also included in the phylogenetic analysis. Six PRs (HsubOR6, HsubOR11, HsubOR13‐16) and HsubOrco from *C. subflexa* were added (Cao et al., 2021). The identified full‐length ORs from *B. mori* (Liu et al., 2014; Tanaka et al., 2009) and *H. assulta* (Jiang et al., 2014; Wu et al., 2019; Xu et al., 2015) were also added.

Four fragmented OBP protein sequences were also removed from each of *H. zea* and *C. virescens* prior to phylogenetic analysis of the OBP gene family, as the protein translations did not start with methionine (M). The identified full‐length OBPs from *B. mori* (Gong et al., 2009; Vieira & Rozas, 2011) and *H. assulta* (Chang et al., 2017; Li, Yang, et al., 2009; Sun et al., 2012) were used for analysis. Because no PBPs were annotated from the reference genomes of *H. armigera* and *H. zea*, publicly available protein sequences of *H. armigera* PBP 1–3 (GenBank accession numbers: CAC08212.1, ACD01993.1, AAO16091.1) and *H. zea* PBP (GenBank accession number: AAC36315.1) were identified and downloaded from GenBank for the phylogenetic analysis. The full list of OR/OBP proteins used for phylogenetic analysis can be found in the Supporting Information.

The protein sequences of the selected ORs and OBPs were aligned using the MUSCLE algorithm (Edgar, 2004), and a maximum likelihood phylogenetic tree was constructed by IQ‐TREE version 3.0.1 (Nguyen et al., 2015) using VT amino acid substitution model. To infer node support, 1000 bootstrap replicates were calculated. The resultant tree was plotted using iTOL (https://itol.embl.de/; Letunic & Bork, 2021). Potential gene duplication and loss events were identified from the trees according to the number of corresponding orthologues, under the assumption that every species should have one orthologue for each gene.

### Gene tree‐species tree reconciliation

To reconstruct the evolutionary history of OR and OBP gene families and distinguish true evolutionary events from phylogenetic noise, we employed the probabilistic reconciliation tool GeneRax v2.0 (Morel et al., 2020). Chemosensory genes from *C. subflexa* and *H. assulta* were excluded from the analysis, as no systematic, genome‐wide manual curation was performed for these species. The Heliothinae species phylogeny described in Cho et al. (2008) was used as the species tree. We utilized the UndatedDTL probabilistic model, which accounts for Duplication, Transfer and Loss (DTL) events while correcting gene tree topology using the Subtree Pruning and Regrafting (SPR) strategy. This approach reconciled the gene trees with the species tree to minimize the joint likelihood of sequence evolution and reconciliation costs. The resulting reconciled trees were visualized using iTOL (https://itol.embl.de/; Letunic & Bork, 2021) and analysed to quantify the number of gene duplications, losses and transfers along each branch of the species phylogeny.

### Read depth estimation of OR and OBP genes

To estimate the read depth of OR and OBP genes, the paired‐end libraries used in de novo assembly were aligned to the reference genome of *C. virescens* (GenBank assembly accession: GCA_002382865.1; Fritz et al., 2018) using Bowtie2 version 2.5.1 (Langmead & Salzberg, 2012) with default parameters. The resulting SAM files were converted to BAM files and sorted using samtools version 1.10 (Li, Handsaker, et al., 2009). The read depth of each gene was measured by samtools depth, and both the average and standard deviation were calculated across the length of each gene.

### Identification of conserved domains and motif composition analysis

For each OR and OBP gene, the amino acid sequence was used to identify conserved domains on the conserved domain database (CDD) of NCBI (https://www.ncbi.nlm.nih.gov/Structure/bwrpsb/bwrpsb.cgi; accessed on 1 December 2024). The protein motifs were discovered using the MEME (version 5.5.7; Bailey et al., 2009) online server with classic mode (https://meme-suite.org/meme/tools/meme). A zoops (Zero or One Occurrence per Sequence) distribution pattern was employed, and the five most significant motifs were obtained with a minimum width of six and a maximum width of 50. The search was performed using the default expectation‐maximization settings with a maximum of 1000 iterations. Background amino acid frequencies were estimated from the input dataset. The results were visualized using TBtools‐II version 2.154 (Chen et al., 2023).

### Homology modelling of 3D structures

AlphaFold3 (Abramson et al., 2024) was used to generate three‐dimensional (3D) structural models for the OR and OBP proteins with default parameters (https://alphafoldserver.com/; accessed on 7 January 2025). For each protein, the model with the highest predicted confidence was selected to ensure reliable structural inference. The pLDDT score (predicted Local Distance Difference Test) was produced by AlphaFold3 to indicate how reliable each residue of a predicted protein structure is. Next, we identified putative ligand‐binding pockets using P2RANK (Krivák & Hoksza, 2018) via the PRANK server (https://prankweb.cz/; Jendele et al., 2019; accessed on 10 January 2025), retaining only the top‐ranked pockets with the highest probability scores for the subsequent analysis. For each protein family, multiple sequence alignment (MSA) was performed using Clustal Omega (ClustalO; Sievers & Higgins, 2014) on the EMBL‐EBI server (https://www.ebi.ac.uk/jdispatcher/msa/clustalo; accessed on 12 January 2025). Amino acid residues of the predicted binding pockets were mapped onto the MSA. To quantify both pocket‐specific and overall protein conservation, we calculated the Shannon entropy (*H*) per alignment column, where *H* = −∑pi*log_2_(pi), ignoring gap positions. Lower entropy values indicate higher conservation, while higher values reflect greater sequence variability. Because the residues directly involved in ligand binding are often more conserved to maintain functional specificity, especially when the proteins bind to similar compounds, mean entropy values were then compared between pocket and non‐pocket columns, and statistical significance was evaluated by *t*‐test to determine whether the binding pockets exhibited significantly higher or lower variability relative to the rest of the protein.

**TABLE 1:** The olfactory receptor (OR) gene family annotated from the reference genome of *Chloridea virescens*.

HvirOR	HzeaOR	Hz_annotation_id	Ha_annotation_id	HarmOR	Scaffold	Start	End	Direction
OR1	HzeaOR1	HzOG200691	HaOG200691	HarmOR1	NWSH01000176.1	234,098	238,032	−
OR2					NWSH01002961.1	3629	12,151	−
Missing	HzeaOR3	HzOG200973	HaOG200973	HarmOR3				
OR4	HzeaOR4	HzOG200977	HaOG200977	HarmOR4	NWSH01000145.1	114,618	118,386	+
OR5	HzeaOR5	HzOG200938	HaOG200938	HarmOR5	NWSH01002259.1	75,484	84,104	−
OR6	HzeaOR6	HzOG200931	HaOG200931	HarmOR6	NWSH01000007.1	26,260	30,918	+
OR6b	HzeaOR6	HzOG200931	HaOG200931	HarmOR6	NWSH01000007.1	34,794	53,163	+
OR7	HzeaOR7	HzOG200856	HaOG200856	HarmOR7	NWSH01000040.1	33,005	36,729	+
OR8	HzeaOR8	HzOG200985	HaOG200985	HarmOR8	NWSH01006510.1	6253	13,277	−
OR9	HzeaOR9	HzOG200747	HaOG200747	HarmOR9	NWSH01002968.1	417	2775	−
OR10	HzeaOR10	HzOG200920	HaOG200920	HarmOR10	NWSH01007896.1	37,220	39,263	+
Missing	HzeaOR11	HzOG201020	HaOG201020	HarmOR11				
OR12	HzeaOR12	HzOG200855	HaOG200855	HarmOR12	NWSH01000040.1	21,714	24,836	+
OR13					NWSH01001866.1	45,142	49,299	−
Missing	HzeaOR13‐like	HzOG215628	HaOG215628	HarmOR13‐like				
OR14	HzeaOR14	HzOG200928	HaOG200928	HarmOR14	NWSH01000007.1	78,506	83,663	−
OR15	HzeaOR15	HzOG200929	HaOG200929	HarmOR15	NWSH01000007.1	68,291	77,211	+
OR16	HzeaOR16	HzOG200930	HaOG200930	HarmOR16	NWSH01000007.1	58,307	64,441	+
OR17	HzeaOR17	HzOG200971	HaOG200971	HarmOR17	NWSH01002272.1	11,811	24,656	+
OR18	HzeaOR18	HzOG200915	HaOG200915	HarmOR18	NWSH01003453.1	3621	6057	+
OR19	HzeaOR19	HzOG200978	HaOG200978	HarmOR19	NWSH01004225.1	12,716	18,929	−
OR20	HzeaOR20	HzOG200982	HaOG200982	HarmOR20	NWSH01000786.1	17,346	24,481	+
OR21	HzeaOR21	HzOG200944	HaOG200944	HarmOR21	NWSH01002810.1	13,233	15,497	+
OR22	HzeaOR22	HzOG200939	HaOG200939	HarmOR22	NWSH01002259.1	85,092	90,794	−
OR23	HzeaOR23	HzOG200904	HaOG200904	HarmOR23	NWSH01005468.1	36,874	42,304	−
OR24	HzeaOR24	HzOG200972	HaOG200972	HarmOR24	NWSH01002098.1	10,225	11,881	+
Missing	HzeaOR25	HzOG200988	HaOG200988	HarmOR25				
OR26	HzeaOR26	HzOG200850	HaOG200850	HarmOR26	NWSH01001847.1	38,534	41,144	+
OR27	HzeaOR27	HzOG200861	HaOG200861	HarmOR27	NWSH01001630.1	31,210	35,701	+
OR28	HzeaOR28	HzOG200822	HaOG200822	HarmOR28	NWSH01002174.1	17,780	20,561	+
OR29	HzeaOR29	HzOG200984	HaOG200984	HarmOR29	NWSH01005276.1	1444	3487	+
OR30	HzeaOR30	HzOG200919	HaOG200919	HarmOR30	NWSH01007896.1	30,882	33,394	+
Missing	HzeaOR31	HzOG200779	HaOG200779	HarmOR31				
OR32	HzeaOR32	HzOG201024	HaOG201024	HarmOR32	NWSH01003434.1	24,718	33,623	+
OR33	HzeaOR33	HzOG200821	HaOG200821	HarmOR33	NWSH01002174.1	4726	14,297	+
Missing	HzeaOR34	HzOG200793	HaOG200793	HarmOR34				
OR35	HzeaOR35	HzOG200880	HaOG200880	HarmOR35	NWSH01003963.1	15,963	20,481	+
OR36	HzeaOR36	HzOG200946	HaOG200946	HarmOR36	NWSH01002232.1	5412	9570	−
OR37	HzeaOR37	HzOG200979	HaOG200979	HarmOR37	NWSH01000780.1	173,644	179,639	−
OR38	HzeaOR38	HzOG200917	HaOG200917	HarmOR38	NWSH01001579.1	123,876	125,677	+
Missing	HzeaOR39	HzOG200916	HaOG200916	HarmOR39				
OR40	HzeaOR40	HzOG200839	HaOG200839	HarmOR40	NWSH01004938.1	9882	18,813	+
OR41	HzeaOR41	HzOG200827	HaOG200827	HarmOR41	NWSH01001711.1	36,170	42,596	+
OR41‐like	HzeaOR41‐like	HzOG212112	HaOG212112	HarmOR41‐like	NWSH01003130.1	35,532	38,975	+
OR42	HzeaOR42	HzOG200780	HaOG200780	HarmOR42	NWSH01002573.1	44,937	45,844	+
Missing	HzeaOR43	HzOG200797	HaOG200797	HarmOR43				
OR44	HzeaOR44	HzOG201018	HaOG201018	HarmOR44	NWSH01002690.1	20,048	27,274	+
OR45	HzeaOR45	HzOG201021	HaOG201021	HarmOR45	NWSH01000683.1	39,929	61,566	+
OR46	HzeaOR46	HzOG200864	HaOG200864	HarmOR46	NWSH01000975.1	18,821	24,356	+
Missing	HzeaOR47	HzOG200772	HaOG200772	HarmOR47				
Missing	HzeaOR48	HzOG200783	HaOG200783	HarmOR48				
Missing	HzeaOR49	HzOG200943	HaOG200943	HarmOR49				
OR50	HzeaOR50	HzOG200941	HaOG200941	HarmOR50	NWSH01002259.1	94,789	101,740	+
Missing	HzeaOR51	HzOG200927	HaOG200927	HarmOR51				
OR52	HzeaOR52	HzOG200820	HaOG200820	HarmOR52	NWSH01002174.1	11,848	14,297	+
OR53	HzeaOR53	HzOG200932	HaOG200932	HarmOR53	NWSH01006314.1	28,982	38,256	+
OR54	HzeaOR54	HzOG200914	HaOG200914	HarmOR54	NWSH01003519.1	43,342	45,897	−
OR55	HzeaOR55	HzOG200840	HaOG200840	HarmOR55	NWSH01001309.1	13,478	14,547	−
OR56	HzeaOR56	HzOG201026	HaOG201026	HarmOR56	NWSH01000442.1	154,283	158,788	−
OR57	HzeaOR57	HzOG201025	HaOG201025	HarmOR57	NWSH01003434.1	35,521	41,175	+
OR58	HzeaOR58	HzOG200947	HaOG200947	HarmOR58	NWSH01002695.1	10,361	13,284	−
OR59	HzeaOR59	HzOG201019	HaOG201019	HarmOR59	NWSH01002690.1	8461	11,151	+
OR60	HzeaOR60	HzOG200799	HaOG200799	HarmOR60	NWSH01007359.1	10	2473	−
Missing	HzeaOR61	HzOG200940	HaOG200940	HarmOR61				
OR62	HzeaOR62	HzOG200942	HaOG200942	HarmOR62	NWSH01002259.1	92,113	93,819	+
OR63	HzeaOR63	HzOG200798	HaOG200798	HarmOR63	NWSH01002961.1	256	1751	−
OR64	HzeaOR64	HzOG200918	HaOG200918	HarmOR64	NWSH01001579.1	109,385	110,552	+
Missing	HzeaOR65	HzOG208187	HaOG208187	HarmOR65				
OR66	HzeaOR66	HzOG208188	HaOG208188	HarmOR66	NWSH01002174.1	97	4097	+
OR67	HzeaOR67	HzOG204092	HaOG204092	HarmOR67	NWSH01002968.1	4126	6253	−
OR68	HzeaOR68	HzOG201254	HaOG201254	HarmOR68	NWSH01000058.1	45,666	50,843	+
OR69	HzeaOR69	HzOG201234	HaOG201234	HarmOR69	NWSH01000302.1	58,054	63,219	+
OR70	HzeaOR70	HzOG214106	HaOG214106	HarmOR70	NWSH01001752.1	12,007	26,950	+
OR71	HzeaOR71	HzOG204587	HaOG204587	HarmOR71	NWSH01001446.1	1535	10,573	+
OR72	HzeaOR72	HzOG204580	HaOG204580	HarmOR72	NWSH01001574.1	21,579	27,879	+
Missing	HzeaOR73	HzOG206392	HaOG206392	HarmOR73				
Missing	HzeaOR74	HzOG200987	HaOG200987	HarmOR74				
Missing	HzeaOR75	HzOG200989	HaOG200989	HarmOR75				
Missing	HzeaOR76	HzOG200990	HaOG200990	HarmOR76				
Missing	HzeaOR77	HzOG201793	HaOG201793	HarmOR77				
OR78	HzeaOR78	HzOG206527	HaOG206527	HarmOR78	NWSH01006065.1	43,849	46,374	+
Missing	HzeaOR79	HzOG207186	HaOG207186	HarmOR79				
OR80					NWSH01002690.1	12,748	14,293	+
OR81					NWSH01002634.1	245	2165	+
OR82					NWSH01000442.1	142,581	147,694	−
OR83					NWSH01000229.1	6023	23,022	+
OR84					NWSH01003458.1	7387	10,221	−
OR85					NWSH01001625.1	77,355	83,869	+
OR86					NWSH01002690.1	211	3206	+
OR87					NWSH01003458.1	14,488	17,423	−
OR88					NWSH01007896.1	33,450	36,270	+
OR89					NWSH01004177.1	10,494	13,504	−
OR90					NWSH01006537.1	11,301	13,303	−
OR91					NWSH01002810.1	4027	8937	+
OR92					NWSH01002810.1	1200	2602	+
OR93					NWSH01002810.1	6443	8937	+
OR94					NWSH01003581.1	16,312	17,839	+
OR95					NWSH01000145.1	290,063	301,383	−
OR96					NWSH01003458.1	280	2178	−
OR97					NWSH01005768.1	2280	5895	+

## RESULTS

### Genome‐wide identification of OR and OBP genes

In total, we manually annotated 81 ORs (including one Orco; Table 1) and 49 OBPs (Table 2) from the *C. virescens* reference genome (GenBank assembly accession: GCA_002382865.1; Fritz et al., 2018) and named them according to the best matched ORs/OBPs of *H. zea* and *H. armigera* (Pearce et al., 2017). Our results indicated that the OBP genes were clustered in the reference genome of *C. virescens*, and at multi‐OBP loci, OBPs were frequently arrayed sequentially and often oriented in the same direction. For example, the 49 OBPs were distributed across 15 scaffolds (of 8826 scaffolds), and almost half of them (24 out of 49) were clustered within a 100 kb region of scaffold NWSH01000044.1 (Figure S1).

### OBP classification

**FIGURE 1:**
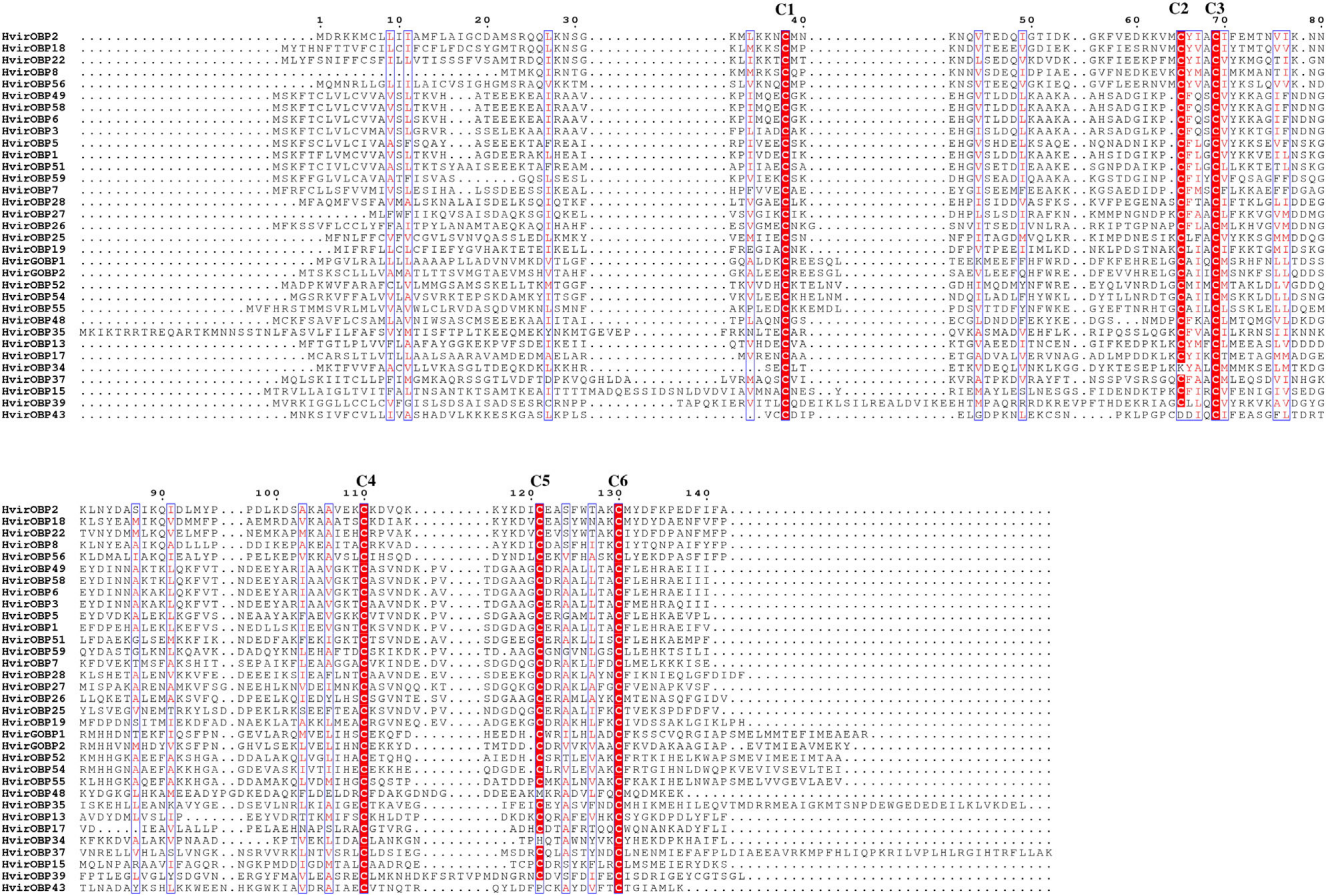
Alignment of the full‐length classical odorant‐binding proteins (OBPs) of *Chloridea virescens*. The protein sequences were aligned by ClustalW. Conserved residues are shown in red, and the six highly conserved cysteines are highlighted as red and marked as C1–C6.

Insect OBPs are divergent, and the overall pairwise sequence similarity is modest (Hekmat‐Scafe et al., 2002). The spacing pattern of the six conserved cysteines is the most typical feature of classical insect OBPs and has been described in *Drosophila* and *B. mori* (Gong et al., 2009; Hekmat‐Scafe et al., 2002; Vieira et al., 2007; Zhou et al., 2004). Our alignment of the *C. virescens* full‐length classical OBP proteins showed the expected pattern and positions of the six conserved cysteines (Figure 1). Following the naming proposed by Hekmat‐Scafe et al. (2002), we identified three types of OBPs in *C. virescens*: 34 Classic OBPs that have the canonical six‐cysteine pattern, eight Minus‐C and seven Plus‐C OBPs that have fewer or more than six conserved cysteine residues, respectively (Table 2). Plus‐C OBPs are atypical OBPs or ‘dimer’ OBPs, which usually contain two domains that are homologous to the domain of Classic OBPs (Manoharan et al., 2013; Vieira & Rozas, 2011).

**TABLE 2:** The OBP gene family annotated from the reference genome of *Chloridea virescens* and their classifications.

HvirOBP	HzeaOBP	Hz_annotation_id	Ha_annotation_id	HarmOBP	Scaffold	Start	End	Direction	Length (aa)	Type
GOBP1	HzeaGOBP1	HzOG200773	HaOG200773	HarmGOBP1	NWSH01003619.1	7268	7915	−	166	Classic
GOBP2	HzeaGOBP2	HzOG200774	HaOG200774	HarmGOBP2	NWSH01000361.1	112,098	114,160	+	164	Classic
OBP1	HzeaOBP1	HzOG200804	HaOG200804	HarmOBP1	NWSH01000044.1	115,144	117,327	+	149	Classic
OBP2	HzeaOBP2	HzOG200790	HaOG200790	HarmOBP2	NWSH01000044.1	74,180	75,575	+	144	Classic
OBP3	HzeaOBP3	HzOG200802	HaOG200802	HarmOBP3	NWSH01000044.1	96,594	97,721	+	149	Classic
OBP4	HzeaOBP4	HzOG200806	HaOG200806	HarmOBP4	NWSH01000044.1	140,536	141,903	+	116	Minus‐C
OBP5	HzeaOBP5	HzOG200805	HaOG200805	HarmOBP5	NWSH01000044.1	130,415	138,664	+	149	Classic
OBP6	HzeaOBP6	HzOG200803	HaOG200803	HarmOBP6	NWSH01000044.1	100,824	105,689	+	149	Classic
OBP7	HzeaOBP7	HzOG200791	HaOG200791	HarmOBP7	NWSH01000044.1	79,096	80,676	+	150	Classic
OBP8	HzeaOBP8	HzOG200787	HaOG200787	HarmOBP8	NWSH01000044.1	61,446	62,694	−	123	Classic
OBP10	HzeaOBP10	HzOG200809	HaOG200809	HarmOBP10	NWSH01000044.1	157,750	160,797	+	138	Minus‐C
OBP12	HzeaOBP12	HzOG200644	HaOG200644	HarmOBP12	NWSH01004598.1	21,615	23,142	−	153	PlusC
OBP13	HzeaOBP13	HzOG200969	HaOG200969	HarmOBP13	NWSH01000208.1	182,287	185,013	+	143	Classic
OBP14	HzeaOBP14	HzOG200841	HaOG200841	HarmOBP14	NWSH01008731.1	3203	3811	+	121	Minus‐C
OBP15	HzeaOBP15	HzOG200853	HaOG200853	HarmOBP15	NWSH01001428.1	67,007	68,231	+	170	Classic
Missing	HzeaOBP16	HzOG200643	HaOG200643	HarmOBP16						
OBP17	HzeaOBP17	HzOG200863	HaOG200863	HarmOBP17	NWSH01001889.1	3640	4149	+	139	Classic
OBP18	HzeaOBP18	HzOG200788	HaOG200788	HarmOBP18	NWSH01000044.1	67,705	71,824	+	148	Classic
OBP19	HzeaOBP19	HzOG200786	HaOG200786	HarmOBP19	NWSH01000044.1	52,149	53,682	−	151	Classic
OBP22	HzeaOBP22	HzOG200789	HaOG200789	HarmOBP22	NWSH01000044.1	72,170	73,555	+	148	Classic
OBP25	HzeaOBP25	HzOG200808	HaOG200808	HarmOBP25	NWSH01000044.1	154,235	157,424	+	149	Classic
OBP26	HzeaOBP26	HzOG200807	HaOG200807	HarmOBP26	NWSH01000044.1	147,121	153,027	−	156	Classic
OBP27	HzeaOBP27	HzOG200800	HaOG200800	HarmOBP27	NWSH01000044.1	85,990	88,126	+	143	Classic
OBP28	HzeaOBP28	HzOG200801	HaOG200801	HarmOBP28	NWSH01000044.1	90,124	92,889	−	154	Classic
Missing	HzeaOBP31	HzOG200796	HaOG200796	HarmOBP31						
OBP32	HzeaOBP32	HzOG200781	HaOG200781	HarmOBP32	NWSH01003491.1	13,897	17,386	+	228	PlusC
OBP33	HzeaOBP33	HzOG200967	HaOG200967	HarmOBP33	NWSH01000549.1	89,636	91,519	−	145	Minus‐C
OBP34	HzeaOBP34	HzOG200966	HaOG200966	HarmOBP34	NWSH01000549.1	93,722	96,167	−	137	Minus‐C
OBP35	HzeaOBP35	HzOG211753	HaOG211753	HarmOBP35	NWSH01000509.1	96,400	97,035	+	214	Classic
OBP36	HzeaOBP36	HzOG200881	HaOG200881	HarmOBP36	NWSH01000018.1	120,612	122,013	−	174	Classic
OBP37	HzeaOBP37	HzOG200925	HaOG200925	HarmOBP37	NWSH01006551.1	16,547	22,213	+	194	Classic
Missing	HzeaOBP38	HzOG200818	HaOG200818	HarmOBP38						
OBP39	HzeaOBP39	HzOG201016	HaOG201016	HarmOBP39	NWSH01000610.1	40,226	74,140	−	187	Classic
Missing	HzeaOBP41	HzOG200642	HaOG200642	HarmOBP41						
OBP42	HzeaOBP42	HzOG200636	HaOG200636	HarmOBP42	NWSH01006041.1	2006	4853	−	203	PlusC
OBP43	HzeaOBP43	HzOG200638	HaOG200638	HarmOBP43	NWSH01006041.1	10,877	12,402	−	139	Classic
Missing	HzeaOBP44	HzOG200640	HaOG200640	HarmOBP44						
OBP45	HzeaOBP45	HzOG201656	HaOG201656	HarmOBP45	NWSH01002197.1	107,188	108,457	−	151	PlusC
Missing	HzeaOBP46	HzOG201657	HaOG201657	HarmOBP46						
OBP47	HzeaOBP47	HzOG206450	HaOG206450	HarmOBP47	NWSH01000044.1	49,758	50,650	−	133	Classic
OBP48					NWSH01000044.1	83,837	85,196	+	150	Classic
OBP49					NWSH01000044.1	109,391	110,854	+	149	Classic
OBP50					NWSH01000044.1	137,478	138,664	+	90	Minus‐C
OBP51					NWSH01000044.1	145,015	146,916	+	152	Classic
OBP52					NWSH01000361.1	130,050	131,132	−	167	Classic
OBP53					NWSH01000361.1	121,965	123,080	+	123	Minus‐C
OBP54					NWSH01000361.1	125,279	126,082	+	166	Classic
OBP55					NWSH01000361.1	119,429	120,561	+	172	Classic
OBP56					NWSH01000044.1	70,164	71,824	+	144	Classic
OBP57					NWSH01006551.1	2037	2509	+	105	Minus‐C
OBP58					NWSH01000044.1	111,807	113,195	+	149	Classic
OBP59					NWSH01000044.1	118,033	120,613	+	144	Classic
OBP60					NWSH01004598.1	5443	7092	−	164	PlusC
OBP61					NWSH01004598.1	1685	4806	−	179	PlusC
OBP62					NWSH01004598.1	11,866	13,459	−	180	PlusC

### Genetic divergence from phylogenetic analysis

**FIGURE 2:**
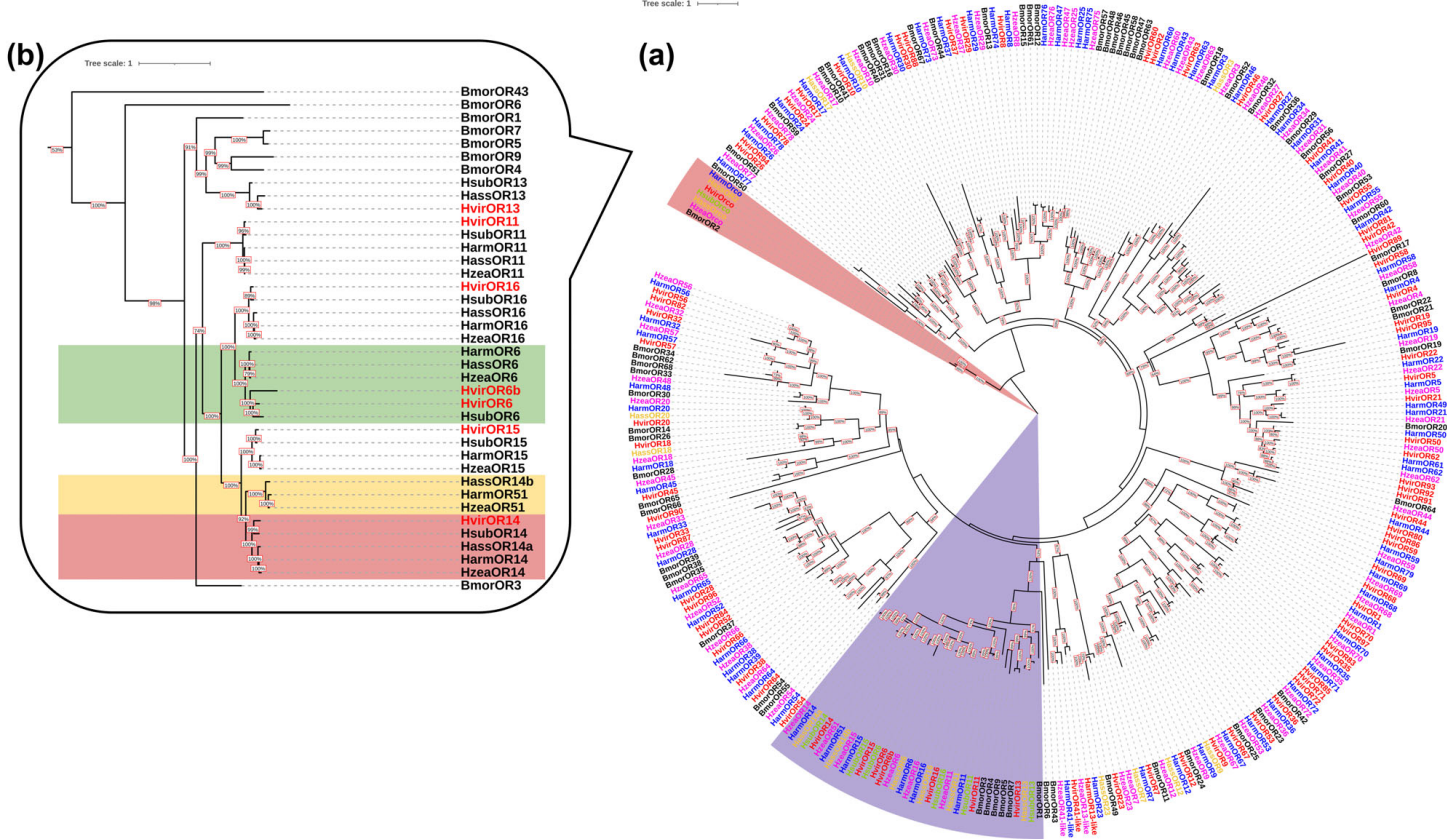
Unrooted maximum likelihood phylogenetic tree of *Chloridea virescens* olfactory receptors (ORs) with other Lepidoptera ORs. Tree scales represent the number of amino acid substitutions per site. (a) Phylogenetic tree based on OR protein sequences from *C. virescens*, *Chloridea subflexa*, *Helicoverpa zea*, *Helicoverpa armigera*, *Helicoverpa assulta* and *Bombyx mori*. The *C. virescens* (red), *C. subflexa* (green), *H. zea* (pink), *H. armigera* (blue), *H. assulta* (yellow) members are shown. The pheromone receptor (PR) and Orco clades are highlighted in light purple and light red, respectively. (b) Phylogenetic tree based on PR protein sequences from *C. virescens*, *C. subflexa*, *H. zea*, *H. armigera*, *H. assulta* and *B. mori*. Red: *C. virescens*. The clades of OR6, OR14a and OR14b are highlighted as light green, light red and light yellow, respectively. The bootstrap values of the branches are indicated on the nodes. Hvir: *C. virescens*, Hsub: *C. subflexa*, Hzea: *H. zea*, Harm: *H. armigera*, Hass: *H. assulta*, Bmor: *B. mori*.

The phylogenetic trees of ORs and OBPs were constructed using the maximum likelihood method with 1000 bootstraps. In the phylogenetic tree of ORs, the Orco proteins were clustered as expected (Figure 2a, highlighted in light red). Orco is a special OR (also known as OR83b) that is highly conserved in most insect species. In addition, PRs from the Heliothine species were clustered with, but were distantly related to, *B. mori* ORs (Figure 2a, highlighted in light purple and Figure 2b). This suggests that distinct gene expansion and divergence events occurred in the Heliothine species and *B. mori*. A recent gene duplication of OR6 was identified in *C. virescens*, with the two tandem copies having very similar protein sequence identities (86.48%).

**FIGURE 3:**
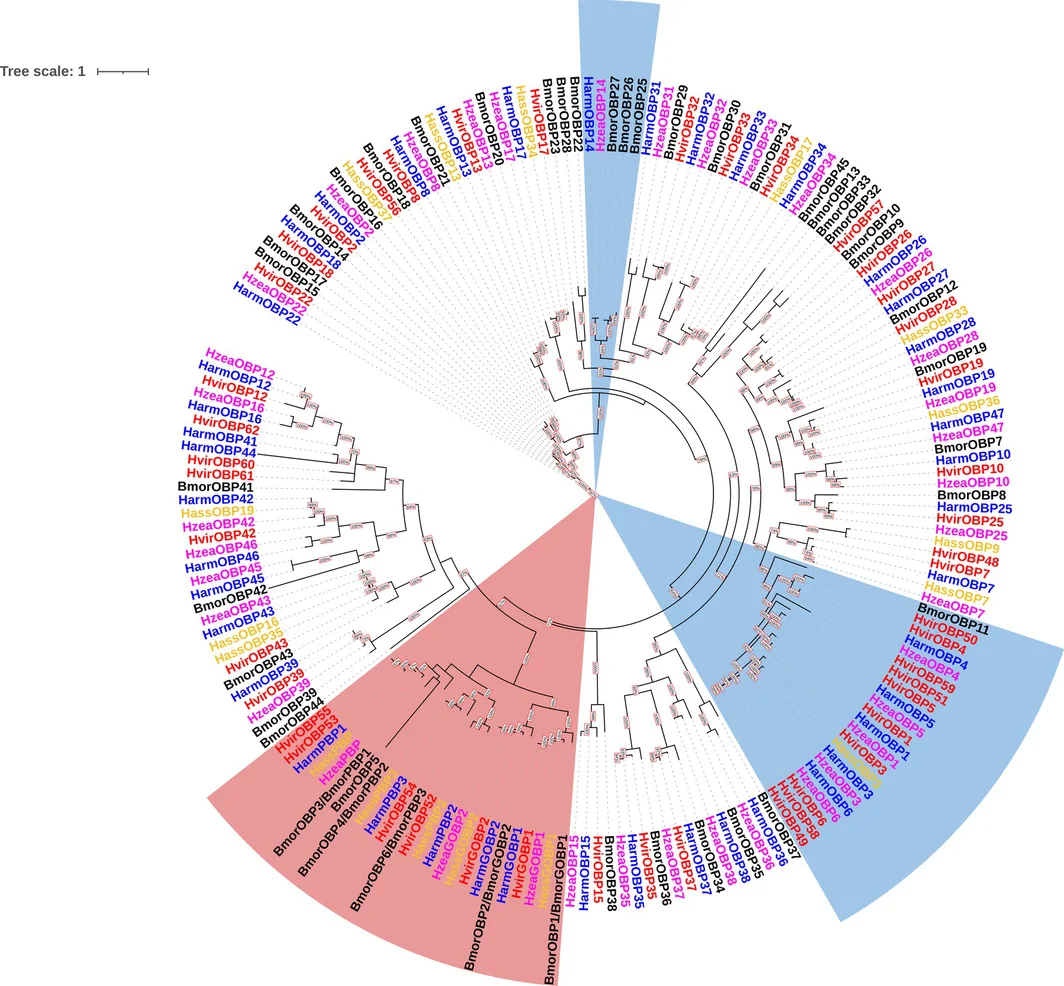
Unrooted maximum likelihood phylogenetic tree based on odorant‐binding protein (OBP) protein sequences from *Chloridea virescens* (red), *Helicoverpa zea* (pink), *Helicoverpa armigera* (blue), *Helicoverpa assulta* (yellow) and *Bombyx mori*. Tree scale represents the number of amino acid substitutions per site. The pheromone‐binding protein (PBP)/general odorant‐binding protein (GOBP) clade is highlighted in light red, and two gene diversification events are highlighted in light blue. The bootstrap values of the branches are indicated on the nodes. Hvir: *C. virescens*, Hzea: *H. zea*, Harm: *H. armigera*, Hass: *H. assulta*, Bmor: *B. mori*.

In the phylogenetic tree of OBPs, PBPs and GOBPs were placed in the same clade (Figure 3, highlighted in light red), which was also observed in *B. mori* (Gong et al., 2009). Our results also indicated a gene expansion in the ‘Minus‐C’ group of *B. mori* (OBP 25–27) (Figure 3, highlighted in light blue). All of them were clustered in a small region of chromosome 5 in the genome of *B. mori* (Gong et al., 2009), indicating a recent rapid gene expansion and diversification. Another rapid gene expansion was observed in the Heliothine species, next to OBP11 of *B. mori* (Figure 3, highlighted in light blue). The OBPs from Heliothine species were more closely related than *B. mori*'*s* OBP, which suggests a gene expansion and diversification in the Heliothinae. Among them, HvirOBP50 (a unique OBP in *C. virescens*) and HvirOBP4 were next to each other in the genome, indicating a recent gene duplication in *C. virescens*.

Phylogenetic reconciliation using GeneRax (Morel et al., 2020) revealed a high rate of gene duplication in *C. virescens*, including 17 duplications in ORs and 6 in OBPs (Tables S2 and S3). Notably, 7 of the 17 OR duplications and 5 of the 6 OBP duplications involved genes located on the same scaffold and in adjacent positions, indicative of recent tandem duplications (highlighted in yellow in Figures S2 and S3). This lineage‐specific expansion coincides with the expansion of host plant use to Solanaceae in *C. virescens*, suggesting a potential association between chemosensory gene expansion and adaptation to diverse host plants.

### Read depth estimation of OR and OBP genes

We estimated read depth of the identified OR and OBP genes to test whether putative gene duplications represent true gene duplications or instead arise from haplotype‐specific alternative contigs (Tables S4 and S5). The average read depth of the whole genome was 54.75. The overall read depth of OR genes was 55.91 ± 18.57, and that of OBP genes was 58.65 ± 11.03, indicating that most of the identified chemosensory genes were true gene duplications. However, five ORs (HvirOR30, HvirOR33, HvirOR70, HvirOR88 and HvirOR90) and two OBPs (HvirOBP6 and HvirOBP50) only had approximately half of the expected coverage (average read depth < 30×), suggesting that they may come from the alternative contigs.

### Identification of conserved domains and motifs

**FIGURE 4:**
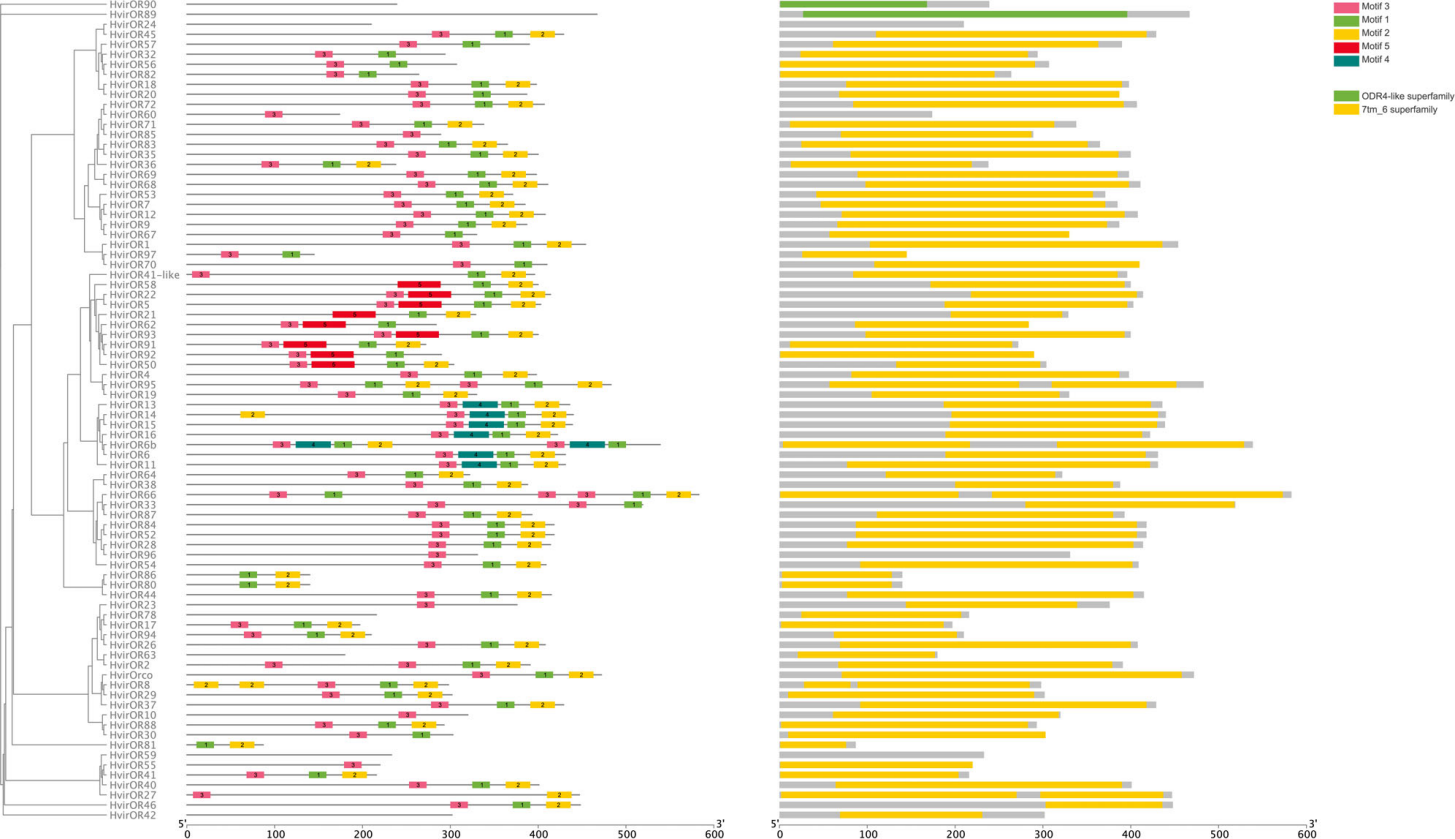
Conserved domains and motifs in *Chloridea virescens* olfactory receptors (ORs) via NCBI‐CDD and MEME. Five significant enriched ungapped motifs were searched in the protein sequences. For the consensus sequences of the motifs, please see Figure S4.

Analysis for the presence of conserved domains via NCBI‐CDD indicated the presence of a ~360 amino acid 7tm_6 superfamily domain in almost all (80 of 82) OR protein sequences (Figure 4). The domain with Pfam ID 7tm_6 is present in seven transmembrane receptors that are most likely *Drosophila* ORs (Paysan‐Lafosse et al., 2025). Furthermore, five significantly enriched ungapped motifs were searched in the OR protein sequences (Figure 4). Our results showed that three of the five motifs appeared more frequently and were mostly located towards the end of the protein sequences. The numbers of sites contributing to the construction of the three motifs were 68, 56 and 74 for motifs 1, 2 and 3, respectively, with a width of 21, 29 and 21 amino acids, respectively (Figure S4). We also found two ‘clade‐specific’ motifs: motif 4 only showed up in the PR clade, and motif 5 was unique in the clade HvirOR58‐HvirOR50; these two motifs may be associated with different types of odorants.

**FIGURE 5:**
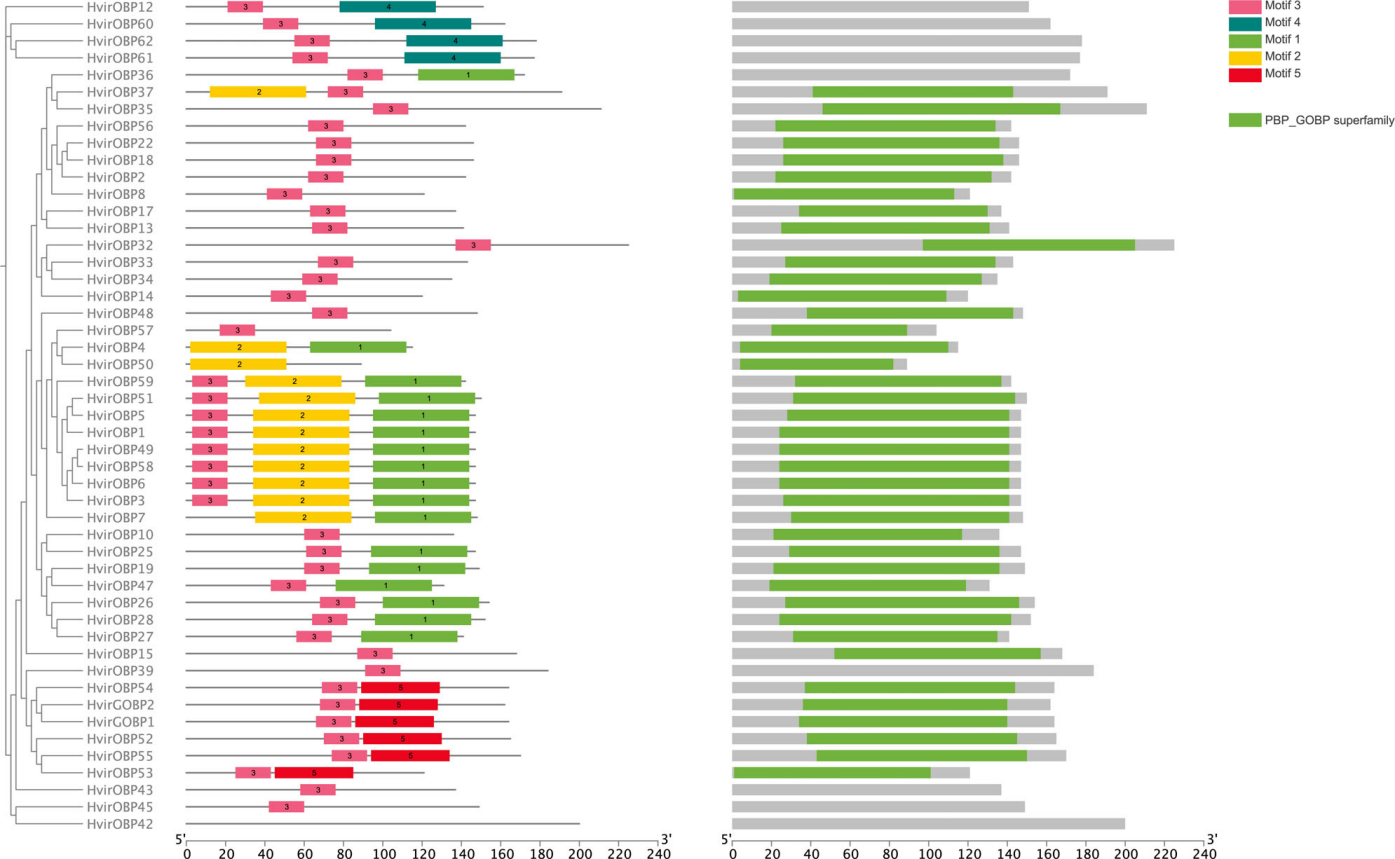
Conserved domains and motifs in *Chloridea virescens* odorant‐binding proteins (OBPs) via NCBI‐CDD and MEME. Five significant enriched ungapped motifs were searched in the protein sequences. For the consensus sequences of the motifs, please see Figure S5.

The top five most significant motifs were also identified from the OBP protein sequences (Figure 5, Figure S5). Our results indicated that OBPs in the same clade tended to have the same motifs, and a possible reason is that these OBPs are involved in binding molecules with similar structures. For example, the ‘GOBP clade’ had motifs 3 and 5, and the ‘Plus‐C clade’, including HvirOBP12, HvirOBP60‐62, had motifs 3 and 4. A PBP_GOBP superfamily domain was commonly found in OBPs (40 of 49), except for the Plus‐C OBPs (also found in HvirOBP32), implying their different functions in odorant detection.

### Homology modelling and binding site prediction

**FIGURE 6:**
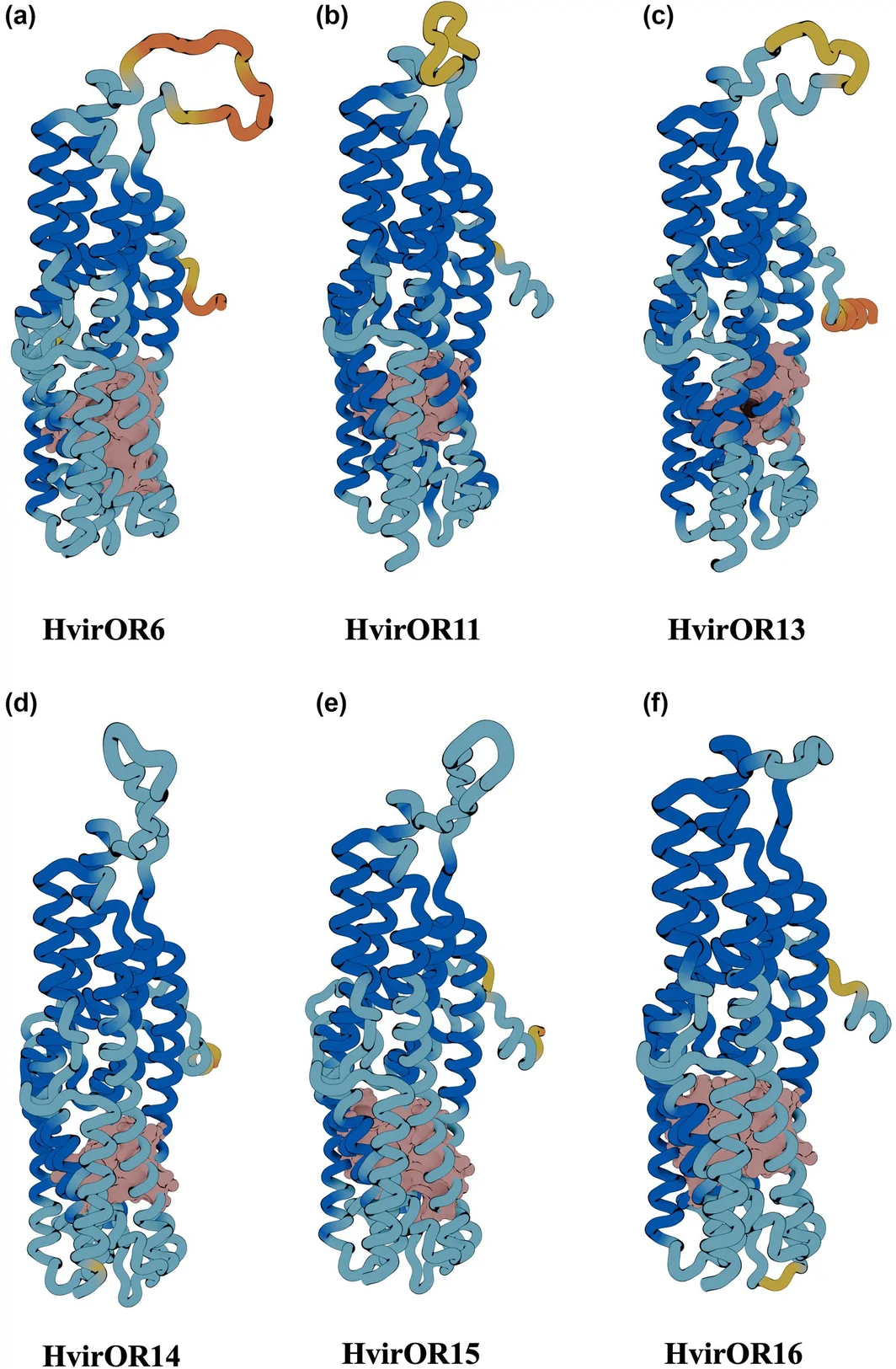
Homology modelling and binding site prediction of pheromone receptors (a) HvirOR6, (b) HvirOR11, (c) HvirOR13, (d) HvirOR14, (e) HvirOR15, (f) HvirOR16 in *Chloridea virescens*, where the colours correspond to different confidence scores from AlphaFold3 (dark blue: pLDDT > 90; blue: 90 > pLDDT > 70; yellow: 70 > pLDDT > 50; orange: pLDDT < 50). The binding pockets are coloured in red.

AlphaFold3 (Abramson et al., 2024) was applied to predict three‐dimensional (3D) structures of the two protein families, and only the highest‐confidence models were selected for binding site predictions using P2RANK (Jendele et al., 2019; Krivák & Hoksza, 2018). The predictions showed that some closely related members share relatively similar binding pockets, implying their potential roles in the detection of molecules with similar structures. Our results were consistent with the phylogenetic analysis. For example, HvirOR1 shares over 50% identity of the binding pocket with its neighbour HvirOR70; two closely related ORs, HvirOR30 and HvirOR88, also have more than 50% identity in their predicted binding pockets. In Figure 6, we showed the predicted structures and binding pockets of a few OR proteins that are involved in pheromone detection in *C. virescens*, where the colour corresponds to different confident scores (dark blue: pLDDT > 90; blue: 90 > pLDDT > 70; yellow: 70 > pLDDT > 50; orange: pLDDT < 50). These ORs shared four conserved motifs (motifs 1–4), and motif 4, which was a motif that only appeared in the PR clade, was predicted as key residues of the binding sites (Table S1). In particular, the neutral residues isoleucine (I), leucine (L) and glutamine (Q) of motif 4 were key residues of the binding pocket, mostly with a motif: LXXILXXQ.

For each gene family, amino acids of the predicted binding pockets were mapped to the MSA to quantify pocket‐specific and whole‐protein conservation, both assessed by Shannon entropy. For ORs, the mean Shannon entropy of the pocket region was 2.537, compared to 0.987 for the non‐pocket region (*p*‐value = 8.309 × 10^−36^). The mean Shannon entropy of the pocket region in OBPs was 2.517, and that of the non‐pocket region was 1.408, with a *p*‐value of 9.489 × 10^−54^ (Figure S6). The statistical significance demonstrated the diversification of the predicted binding sites rather than a rigid, highly conserved one, which is not surprising as these chemosensory proteins are involved in various volatile detection.

## DISCUSSION

Antennal transcriptome data have been widely used to identify chemosensory genes due to its low cost and relatively easy data generation (Dong et al., 2023; Hu et al., 2022; Jiang et al., 2021; Yang et al., 2024; Zhang, Wang, et al., 2015). However, transcriptome‐based annotations may underestimate the total number of genes compared to genome‐wide identification, and one reason is that lowly expressed genes and pseudogenes are often not captured. In the study, we leveraged the structural annotations and existing evidence from transcriptome and protein analyses, to annotate 81 ORs, including one Orco, as well as 49 OBPs from the *C. virescens* reference genome. Our results showed that OBP genes were highly clustered in the reference genome of *C. virescens*, but OR genes appeared more scattered throughout the genome, having only a few clusters. The chromosomal clustering of OBPs was first observed in *Drosophila melanogaster* (Hekmat‐Scafe et al., 2002; Vogt et al., 2002) and is believed to be associated with the expression and regulation of OBPs (Vogt et al., 2002). Across the genomes of 12 *Drosophila* species, 69% of OBP genes are arranged in 10 clusters (Vieira et al., 2007). In *B. mori*, the OBP genes are not randomly distributed in the genome, and most of them appear to be in close physical proximity within a specific region, for example, 20 OBPs are clustered on five chromosomes (Gong et al., 2009). The same clustering distribution of OBPs was also observed in mosquitos: *Anopheles gambiae* (Xu et al., 2003), *Anopheles sinensis* (He et al., 2016), *Culex quinquefasciatus* (Ryazansky et al., 2024). Previous research showed that OBP genes in the same cluster can be co‐expressed in adult olfactory sensilla, suggesting that the clustering of these genes is linked to their co‐regulation (Vogt et al., 2002). An explanation for the multiple, widely dispersed gene clusters of *C. virescens* ORs is that inter‐chromosomal translocation events are more frequent in ORs than OBPs (Conceição & Aguadé, 2008).

*Chloridea virescens* has more identified OBPs than other annotated Heliothine species: *H. zea* and *H. armigera* both have 40 OBPs in their reference genomes (Pearce et al., 2017). A model species in Lepidoptera, *B. mori*, has 44 identified OBPs and 43 of them are full‐length (Gong et al., 2009; Vieira & Rozas, 2011). One possible reason for the increase in *C. virescens* is the different qualities of their reference genomes. The reference genome of *C. virescens* is more fragmented than the other two *Helicoverpa* species, and considering the estimated read depth of OBP genes, HvirOBP6 and HvirOBP50 may not reflect true gene duplication events but rather come from alternative haplotype‐specific contigs. The highly fragmented assembly raises the possibility that haplotypic duplications may inflate gene counts. Although we performed read depth analyses to help distinguish true gene duplications from assembly artefacts, this limitation should still be considered in future analysis.

Phylogenetic analyses revealed expected clustering patterns. Orco genes are clustered in the OR tree as expected, due to their high similarity and conservation across insect species. PRs from different Heliothine species also cluster together. This was expected because females of different Heliothine species create their pheromone blends with some of the same volatile compounds used in different ratios. Males can respond to the pheromone compounds, but the strength of the response may vary according to the corresponding physiological or functional assays (Cao et al., 2023; Jiang et al., 2014; Mo et al., 2023; Wang et al., 2024). Because these PRs can respond to some of the same ligands, there is also some sequence similarity at the protein level. Notably, within the PR clade (Figure 2b), OR14 has two copies in *H. armigera*, *H. zea* and *H. assulta*, but only one copy in the *C. virescens* genome. This pattern may reflect a potential gene loss in *C. virescens* and *C. subflexa*, or a gene duplication in the *Helicoverpa* species. Our results also showed a recent gene duplication of OR6 in *C. virescens*, and the two copies are located adjacent to each other in the genome. However, considering the fragmented reference genome and limited structural annotation in the region, further validation will be required to confirm the duplication.

The PBP sub‐family of OBPs contains proteins that bind and solubilize the hydrophobic pheromone components and transfer them to the specific PRs. Surprisingly, no PBPs were annotated from *H. armigera* and *H. zea* by Pearce et al. (2017), although previous studies have identified several PBPs in *Helicoverpa* species using RT‐PCR (Callahan et al., 2000; Dong et al., 2017; Guo et al., 2012; Wang et al., 2004; Zhang et al., 2012). Blast analyses confirmed that these PBPs are present in the genomes of *H. armigera* and *H. zea* (GenBank assembly accessions: GCA_002156985.1, GCA_002150865.1) but were not annotated during manual curation; therefore, we manually added HarmPBP 1–3 and HzeaPBP to the phylogenetic analysis. An explanation for the missing PBPs by Pearce et al. (2017) is the quality limitation of the first reference genomes of *H. armigera* and *H. zea*, which were used here because some chemosensory receptor gene families had been manually curated. With a fragmented short‐read reference genome, these genes could be fragmented or missing from the identified genes.

Phylogenetic reconciliation using GeneRax (Morel et al., 2020) revealed a high rate of chemosensory gene expansion in *C. virescens* after speciation, consistent with an adaptive response to the heterogeneous olfactory environments of polyphagous species. Although this approach provides a powerful framework for jointly inferring gene duplication and loss while accounting for the species tree, the limitations of our results need to be considered. In particular, chemosensory gene annotation is highly sensitive to reference genome quality, raising the possibility that some inferred gene duplications and losses may reflect undetected, fragmented or misannotated genes rather than true evolutionary events. Considering the quality of the *C. virescens* genome, we manually examined the identified duplication events within the species, and genes located on the same scaffold and in adjacent positions are more likely to reflect true duplication events.

Gene duplication followed by neo‐ or sub‐functionalization is a well‐recognized mechanism generating molecular and functional diversity in sensory systems (Sánchez‐Gracia et al., 2009). In *Drosophila*, approximately two‐thirds of gene duplications undergo neofunctionalization (Assis & Bachtrog, 2013). Chemosensory gene duplications may be followed by positive selection on the ligand‐binding site (Saad et al., 2018) to detect new host volatiles or pheromones (neofunctionalization), or the duplicated genes may become more specialized in detecting specific odours instead of one gene that responds to several similar odours (sub‐functionalization). In Heliothine species, lineage‐specific expansions of chemosensory gene families may provide a molecular basis for ecological diversification and host adaptation. However, it remains challenging to connect these molecular changes to evolutionary, behavioural and ecological changes, as the functions of most chemosensory genes are unknown.

Due to their large size (>40 kDa) and variability, experimentally resolving the three‐dimensional structures of insect ORs has been extremely challenging (Carraher et al., 2015), and most previous studies focused on understanding OR's structure via transmembrane topology prediction (Cao et al., 2021; Liu et al., 2018; Zhang et al., 2014). In our structural analyses, we used AlphaFold3, which leverages advanced AI to predict high‐resolution, three‐dimensional protein structures and identified key residues involved in ligand‐receptor interactions. We found a ‘PR‐specific’ motif (motif 4) within the binding sites of all PRs, and the motif appears to be under purifying selection, suggesting its functional importance and evolutionary constraint. Our results supported a model that the shared structural element within the binding pockets of different PRs plays an important role in the interactions with diverse pheromone components, and several possible explanations are as below:

1. The motif may play a role in OBP‐OR recognition. While no OBP‐OR recognition models have been validated in invertebrates, Matarazzo et al. (2002) proposed an OBP‐OR direct recognition model in humans. In this model, high‐affinity interaction between OBP and OR suggested that under physiological conditions, ORs may be specifically paired with an OBP partner in the absence of odorant (Matarazzo et al., 2002). However, the recognition model may be unique to humans or vertebrates and may not be the case in insects, as they are evolutionarily divergent.
2. Pheromone recognition across different PRs appears to rely on a conserved binding site (Del Mármol et al., 2021), and the shared motif plays a critical role in maintaining the structural integrity of the PR binding pocket. Recent research has identified diverse motifs among different groups of human ORs, and the motifs can interact with other regions of transmembrane domains, contributing to the stabilization of the receptor's 3D structure (Billesbølle et al., 2023; Ryu et al., 2024).
3. The shared motif may directly contribute to ligand binding by forming hydrogen bonds, polar interactions or other contacts that anchor ligands in the binding pockets. Structural studies have shown that some conserved residues in the binding pocket can generate additional anchoring points for various odorant structures, in line with the receptor's wide range of recognition capabilities (Choi et al., 2023; Comte et al., 2025; Del Mármol et al., 2021).
4. The pocket might have a set of residues that provide a baseline for binding, while amino acid diversification at key positions enables precise recognition of specific pheromone molecules. A recent study in *B. mori* demonstrated that males can distinguish two nearly identical compounds—bombykol and bombykal—through the formation of a reversible covalent bond (Jang et al., 2025). This work proposed a binding mode between PRs and pheromone compounds and showed that a key covalent bond allows moths to discriminate between molecules that differ only in a single hydrogen atom (Jang et al., 2025).

Pheromone‐based control strategies represent a promising approach for pest management through disruption of moth mating behaviour via PR inhibition (Venthur & Zhou, 2018). Although *C. virescens* is effectively controlled by Bt crops in many regions of the world, it remains an important pest in tobacco, where no transgenic variety is available (Blanco, 2012). *Chloridea virescens* serves as a valuable model species for studying related agriculture pests within the Heliothinae sub‐family. Insights gained from *C. virescens* PR structures can be applied to design pheromone disruptors for other economically significant pests, paving the way for next‐generation, precision‐targeted pest management strategies.

## CONCLUSION

In conclusion, we identified 81 ORs (including one Orco) and 49 OBPs from the *C. virescens* reference genome. Three types of OBPs were classified according to the pattern of six conserved cysteines: 34 classic OBPs, 8 Minus‐C OBPs and 7 Plus‐C OBPs. In addition, a phylogenetic analysis was performed to identify potential gene duplication and gene loss of OR and OBP gene families among members of the Heliothinae. Two novel gene expansion events were observed in the ‘Minus‐C’ group of *B. mori* and the Heliothine moths. The gene diversification events could be associated with the differences of odorant detection and olfactory‐mediated behaviours between the Heliothine moths. We also identified a ‘PR‐specific’ motif localized in the predicted binding pocket of PRs, which appears to be under purifying selection and likely plays a critical role in ligand binding and pheromone specificity. Our study provides important insights into the critical features of these PRs to understand pheromone specificity and enables further investigation into how mutations or structural variations in the motif affect ligand binding and, consequently, species‐specific pheromone perception and behaviour.

## AUTHOR CONTRIBUTIONS

**Rong Guo:** Conceptualization; methodology; formal analysis; writing – original draft; writing – review and editing; visualization; software; data curation. **Boyang Ni:** Formal analysis; visualization; software. **Megan L. Fritz:** Conceptualization; methodology; supervision; funding acquisition; writing – review and editing; resources.

## CONFLICT OF INTEREST STATEMENT

The authors declare that they have no conflicts of interest related to the research, authorship or publication of this manuscript.

## Supporting information


**Figure S1.** OBP gene cluster presents in scaffold NWSH01000044.1 (scaffold length = 319,136 bp). Genes are depicted as triangles: red indicates forward transcription (+) and blue indicates reverse transcription (−).

**Figure S2.** The inferred OR gene tree reconciled with the species tree. Nodes are labelled as D (duplication), S (speciation) or T (horizontal gene transfer). Species are coloured as follows: *Chloridea virescens* (pink), *Helicoverpa zea* (green), *Helicoverpa armigera* (purple) and *Bombyx mori* (black). Duplications involving genes located on the same scaffold and in adjacent positions are highlighted in light yellow.

**Figure S3.** The inferred OBP gene tree reconciled with the species tree. Nodes are labelled as D (duplication), S (speciation) or T (horizontal gene transfer). Species are coloured as follows: *Chloridea virescens* (pink), *Helicoverpa zea* (green), *Helicoverpa armigera* (purple) and *Bombyx mori* (black). Duplications involving genes located on the same scaffold and in adjacent positions are highlighted in light yellow.

**Figure S4.** Identification of conserved motifs in HvirORs via MEME.

**Figure S5.** Identification of conserved motifs in HvirOBPs via MEME.

**Figure S6.** The Shannon entropy distribution of the pocket and non‐pocket regions in (A) HvirORs and (B) HvirOBPs.

**Data S1.** HvirORs.

**Data S2.** HvirOR_OBP_cDNA.

**Data S3.** HvOBPs.

**Data S4.** ModelSelection_OBP.

**Data S5.** ModelSelection_OR.

**Data S6.** OBP_phylogenetic.

**Data S7.** OR_phylogenetic.

**Table S1.** Predicted binding site residues of PRs in *Chloridea virescens*.
**Table S2.** The number of diversification events in OR gene family per species, estimated by Generax (Morel et al., 2020).
**Table S3.** The number of diversification events in OBP gene family per species, estimated by Generax (Morel et al., 2020).
**Table S4.** Read depth of OR genes in *Chloridea virescens*.
**Table S5.** Read depth of OBP genes in *Chloridea virescens*.

## Data Availability

The data that support the findings of this study are openly available in the Dryad Digital Repository (Guo et al., 2026) at https://doi.org/10.5061/dryad.qrfj6q5xc.
