# Supplementary material for: Genome‐wide identification of olfactory receptor and odorant‐binding protein gene families and their roles in Heliothine chemosensory evolution
Source: Insect Mol Biol. 2026 Jun 28;35(5):594–612. doi: 10.1111/imb.70052 (PMC13551510; doi:10.1111/imb.70052)
Supplement: Supplementary file 1 [file IMB-35-594-s002.pdf]

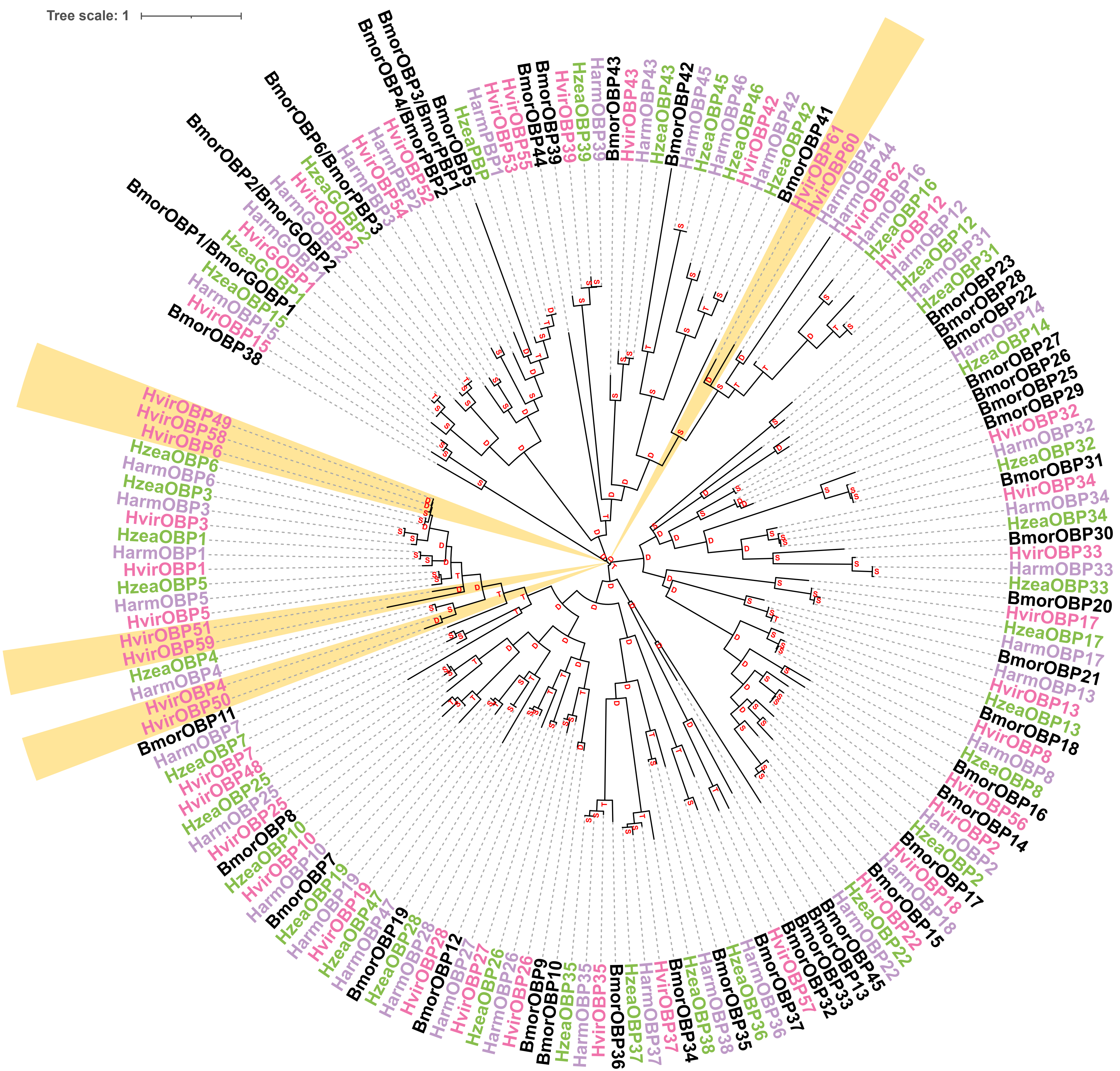
Tree scale: 1
BmorOBP38
HvirOBP15
HarmOBP15
HzeaOBP15
BmorOBP1/BmorGOBP1
HzeaGOBP1
HvirGOBP1
HarmGOBP1
BmorOBP2/BmorGOBP2
HarmGOBP2
HvirGOBP2
HzeaGOBP2
BmorOBP6/BmorPBP3
HarmPBP3
HvirOBP54
HarmPBP2
HvirOBP52
BmorOBP4/BmorPBP2
BmorOBP3/BmorPBP1
BmorOBP5
HzeaPBP
HarmPBP1
HvirOBP53
HvirOBP55
BmorOBP44
BmorOBP39
HvirOBP39
HzeaOBP39
HarmOBP39
BmorOBP43
HvirOBP43
HarmOBP43
HzeaOBP43
BmorOBP42
HarmOBP45
HzeaOBP45
HarmOBP46
HzeaOBP46
HvirOBP42
HarmOBP42
HzeaOBP42
BmorOBP41
HvirOBP61
HvirOBP60
HarmOBP41
HarmOBP44
HvirOBP62
HarmOBP16
HzeaOBP16
HvirOBP12
HarmOBP12
HzeaOBP12
HarmOBP31
HzeaOBP31
BmorOBP23
BmorOBP28
BmorOBP22
HarmOBP14
HzeaOBP14
BmorOBP27
BmorOBP26
BmorOBP25
BmorOBP29
HvirOBP32
HarmOBP32
HzeaOBP32
BmorOBP31
HvirOBP34
HarmOBP34
HzeaOBP34
BmorOBP30
HvirOBP33
HarmOBP33
HzeaOBP33
BmorOBP20
HvirOBP17
HzeaOBP17
HarmOBP17
BmorOBP21
HarmOBP13
HvirOBP13
HzeaOBP13
BmorOBP18
HvirOBP8
HarmOBP8
HzeaOBP8
BmorOBP16
HvirOBP56
BmorOBP14
HvirOBP2
HarmOBP2
HzeaOBP2
BmorOBP17
HvirOBP18
HarmOBP18
BmorOBP15
HzeaOBP22
HvirOBP22
HarmOBP22
BmorOBP45
BmorOBP13
BmorOBP33
BmorOBP32
HvirOBP57
BmorOBP37
HarmOBP36
HzeaOBP36
BmorOBP35
HarmOBP38
HzeaOBP38
BmorOBP34
HvirOBP37
HarmOBP37
HzeaOBP37
BmorOBP36
HvirOBP35
HarmOBP35
HzeaOBP35
BmorOBP10
BmorOBP9
HvirOBP26
HarmOBP26
HzeaOBP26
HvirOBP27
HarmOBP27
BmorOBP12
HvirOBP28
HarmOBP28
HzeaOBP28
BmorOBP19
HarmOBP47
HzeaOBP47
HvirOBP19
HarmOBP19
HzeaOBP19
BmorOBP7
HarmOBP10
HvirOBP10
HzeaOBP10
BmorOBP8
HvirOBP25
HarmOBP25
HzeaOBP25
HvirOBP48
HvirOBP7
HzeaOBP7
HarmOBP7
BmorOBP11
HvirOBP50
HvirOBP4
HarmOBP4
HzeaOBP4
HvirOBP59
HvirOBP51
HvirOBP5
HarmOBP5
HzeaOBP5
HvirOBP1
HarmOBP1
HzeaOBP1
HvirOBP3
HarmOBP3
HzeaOBP3
HarmOBP6
HzeaOBP6
HvirOBP6
HvirOBP58
HvirOBP49
