# Supplementary material for: Genome‐wide identification of olfactory receptor and odorant‐binding protein gene families and their roles in Heliothine chemosensory evolution
Source: Insect Mol Biol. 2026 Jun 28;35(5):594–612. doi: 10.1111/imb.70052 (PMC13551510; doi:10.1111/imb.70052)
Supplement: Supplementary file 2 [file IMB-35-594-s013.pdf]

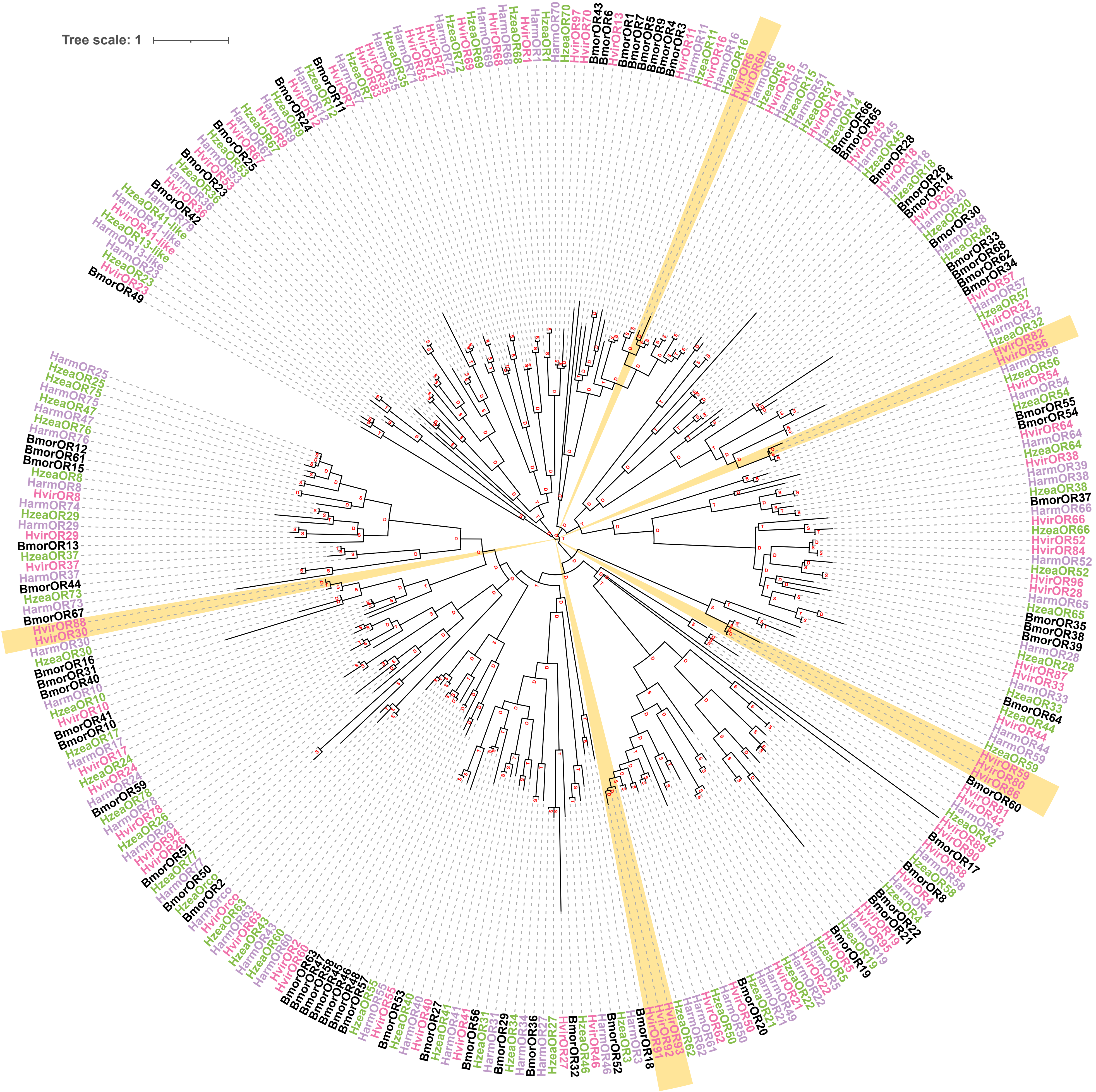
Tree scale: 1
