# Supplementary material for: Genome‐wide identification of olfactory receptor and odorant‐binding protein gene families and their roles in Heliothine chemosensory evolution
Source: Insect Mol Biol. 2026 Jun 28;35(5):594–612. doi: 10.1111/imb.70052 (PMC13551510; doi:10.1111/imb.70052)
Supplement: Supplementary file 3 [file IMB-35-594-s010.docx]

**Supplemental Materials**

**Table S1.** Predicted binding site residues of PRs in *C. virescens*.

| Pheromone receptors | Predicted binding sites |
| --- | --- |
| HvirOR6 | 63ASN, 66THR, 91LEU, 92 ILE, 95ILE, 96GLY, 99ASP, 156GLN, 157MET, 160GLY, 161LEU, 164PHE, 192LEU, 217ILE, 218SER, 221CYS, 222SER, 225PHE, 226CYS, 339LEU, 342ILE, 343MET, 346GLN |
| HvirOR11 | 61TYR, 83GLN, 86ILE, 87VAL, 90MET, 91ASN, 93LEU, 148LEU, 151LEU, 152LEU, 155ALA, 156ALA, 159PHE, 162ILE, 163PRO, 188ILE, 213GLY, 214THR, 217CYS, 218GLY, 221VAL, 222SER, 339ARG, 340TYR, 343LEU, 346ILE, 347MET, 350GLU |
| HvirOR13 | 93THR, 96MET, 97ASN, 153LEU, 156LEU, 157ALA, 160GLY, 164PHE, 192VAL, 217ILE, 218SER, 221CYS, 222THR, 225PHE, 226CYS, 343PRO, 344LEU, 347VAL, 350GLN, 351GLN |
| HvirOR14 | 98ILE, 99SER, 102MET, 103THR, 163GLN, 164MET, 167GLY, 168LEU, 170LEU, 171PHE, 199LEU, 224ILE, 225SER, 228CYS, 229SER, 232PHE, 233CYS, 351PRO, 352LEU, 355ILE, 356LEU, 359GLN |
| HvirOR15 | 66HIS, 69LEU, 70CYS, 73ALA, 98ILE, 99SER, 102MET, 103MET, 106THR, 164MET, 167GLY, 168LEU, 170LEU, 171PHE, 174THR, 199LEU, 224LEU, 225SER, 227THR, 228CYS, 229SER, 232PHE, 233CYS, 347ARG, 350PRO, 351LEU, 354ILE, 355LEU, 358GLN |
| HvirOR16 | 63THR, 66ALA, 92VAL, 95MET, 96SER, 99ASP, 156GLN, 157MET, 160GLY, 161LEU, 163LEU, 164PHE, 192LEU, 194TYR, 217LEU, 218SER, 221CYS, 222SER, 224TRP, 225PHE, 226CYS, 334LEU, 337ILE, 338LEU, 341GLN |

**Table S2. The number of diversification events in OR gene family per species, estimated by Generax (Morel et al, 2020).**

| **Species label** | **Speciations** | **Duplications** | **Losses** | **Transfers** |
| --- | --- | --- | --- | --- |
| Node_Bmor_Harm_0 | 22 | 21 | 0 | 6 |
| Node_Harm_Hzea_0 | 76 | 3 | 0 | 1 |
| Node_Harm_Hvir_0 | 63 | 28 | 0 | 8 |
| Hvir | 82 | 17 | 3 | 9 |
| Hzea | 74 | 0 | 6 | 1 |
| Harm | 80 | 0 | 1 | 1 |
| Bmor | 68 | 37 | 0 | 20 |

**Table S3. The number of diversification events in OBP gene family per species, estimated by Generax (Morel et al, 2020).**

| **Species label** | **Speciations** | **Duplications** | **Losses** | **Transfers** |
| --- | --- | --- | --- | --- |
| Node_Bmor_Harm_0 | 21 | 20 | 0 | 2 |
| Node_Harm_Hzea_0 | 41 | 1 | 1 | 3 |
| Node_Harm_Hvir_0 | 34 | 4 | 0 | 2 |
| Hvir | 45 | 6 | 2 | 5 |
| Hzea | 37 | 0 | 6 | 1 |
| Harm | 43 | 1 | 1 | 1 |
| Bmor | 43 | 20 | 0 | 15 |

**Table S4.** Read depth of OR genes in *C. virescens*.

| **ORs** | **Average** | **SD** | **ORs** | **Average** | **SD** |
| --- | --- | --- | --- | --- | --- |
| OR1 | 61.37 | 8.36 | OR46 | 34.36 | 34.14 |
| OR2 | 40.95 | 26.5 | OR50 | 34.43 | 35.24 |
| OR4 | 67.39 | 10.04 | OR52 | 33.1 | 4.72 |
| OR5 | 47.98 | 64.19 | OR53 | 64.99 | 11.99 |
| OR6 | 65.67 | 7.5 | OR54 | 63.48 | 9.84 |
| OR6b | 58.69 | 31.06 | OR55 | 58.41 | 8.81 |
| OR7 | 68.02 | 8.18 | OR56 | 89.63 | 80.9 |
| OR8 | 49.78 | 30.62 | OR57 | 67.79 | 8.62 |
| OR9 | 65.87 | 6.58 | OR58 | 63.76 | 7.74 |
| OR10 | 71.59 | 8.99 | OR59 | 66.4 | 30.6 |
| OR12 | 81.14 | 26.52 | OR60 | 34.47 | 6.42 |
| OR13 | 70.7 | 17.19 | OR62 | 62.15 | 7 |
| OR14 | 66.07 | 10.92 | OR63 | 53.33 | 20.99 |
| OR15 | 49.85 | 32.29 | OR64 | 64.83 | 6.76 |
| OR16 | 66.02 | 13.65 | OR66 | 39.17 | 16.86 |
| OR17 | 48.31 | 57.67 | OR67 | 69.27 | 7.81 |
| OR18 | 65.89 | 6.93 | OR68 | 69.72 | 8.78 |
| OR19 | 69.82 | 15.09 | OR69 | 56.99 | 18.08 |
| OR20 | 84.37 | 75.27 | OR70 | 29.98 | 20.82 |
| OR21 | 63.21 | 7.52 | OR71 | 67.71 | 27.07 |
| OR22 | 45.59 | 41.04 | OR72 | 68.12 | 10.21 |
| OR23 | 44.35 | 17.11 | OR78 | 41.99 | 17.53 |
| OR24 | 64.88 | 9.53 | OR80 | 54.66 | 19.17 |
| OR26 | 32.09 | 5.37 | OR81 | 54.87 | 14.3 |
| OR27 | 41.44 | 16.58 | OR82 | 70.36 | 31.85 |
| OR28 | 34.54 | 9.73 | OR83 | 60.16 | 36.84 |
| OR29 | 67.07 | 6.71 | OR84 | 31.37 | 5.67 |
| OR30 | 26.77 | 10.46 | OR85 | 64.69 | 9.75 |
| OR32 | 43.78 | 31.57 | OR86 | 48.25 | 13.94 |
| OR33 | 24.91 | 14.95 | OR87 | 36.14 | 5.19 |
| OR35 | 68.85 | 13.66 | OR88 | 29.08 | 13.16 |
| OR36 | 64.67 | 9.65 | OR89 | 42.51 | 17.16 |
| OR37 | 65.48 | 8.53 | OR90 | 29.45 | 10.42 |
| OR38 | 66.17 | 12.64 | OR91 | 63.67 | 8.81 |
| OR40 | 47.47 | 34.42 | OR92 | 64.94 | 6.34 |
| OR41 | 64.9 | 11.55 | OR93 | 66.99 | 9.34 |
| OR41-like | 94.25 | 28.86 | OR94 | 35.87 | 7.52 |
| OR42 | 61.1 | 7.12 | OR95 | 103.5 | 57.5 |
| OR44 | 61.39 | 13.94 | OR96 | 31.72 | 6.2 |
| OR45 | 35.27 | 34.96 | OR97 | 32.97 | 8.07 |

**Table S5.** Read depth of OBP genes in *C. virescens*.

| **OBPs** | **Average** | **SD** | **OBPs** | **Average** | **SD** |
| --- | --- | --- | --- | --- | --- |
| GOBP1 | 60.85 | 7.48 | OBP34 | 60.39 | 7.58 |
| GOBP2 | 67.37 | 9.15 | OBP35 | 65.67 | 6.45 |
| OBP1 | 65.14 | 9.32 | OBP36 | 60.41 | 7.36 |
| OBP2 | 73.57 | 10.09 | OBP37 | 44.09 | 17.38 |
| OBP3 | 62.89 | 8.4 | OBP39 | 60.35 | 23.61 |
| OBP4 | 48.14 | 16.51 | OBP42 | 66.44 | 8.6 |
| OBP5 | 35.7 | 16.02 | OBP43 | 64.8 | 8.76 |
| OBP6 | 20.76 | 30.46 | OBP45 | 48.82 | 16.87 |
| OBP7 | 71.47 | 8.54 | OBP47 | 65.6 | 10.54 |
| OBP8 | 69.4 | 6.25 | OBP48 | 63.05 | 8.5 |
| OBP10 | 67.88 | 9.82 | OBP49 | 35.53 | 7.49 |
| OBP12 | 63.34 | 9.07 | OBP50 | 27.46 | 9.71 |
| OBP13 | 67.63 | 7.44 | OBP51 | 66.37 | 7.02 |
| OBP14 | 49.87 | 8.41 | OBP52 | 67.25 | 6.8 |
| OBP15 | 64 | 7.51 | OBP53 | 32.2 | 5.6 |
| OBP17 | 58.88 | 8.25 | OBP54 | 62.57 | 8.45 |
| OBP18 | 67.13 | 8.18 | OBP55 | 42.97 | 15.36 |
| OBP19 | 65.19 | 11.08 | OBP56 | 65.34 | 8.29 |
| OBP22 | 71.64 | 8.7 | OBP57 | 66.04 | 8.8 |
| OBP25 | 65.15 | 15.93 | OBP58 | 32.64 | 7.29 |
| OBP26 | 34.21 | 38.53 | OBP59 | 67.79 | 8.21 |
| OBP27 | 68.49 | 9.76 | OBP60 | 63.41 | 8.83 |
| OBP28 | 61.31 | 14.07 | OBP61 | 68.78 | 15.96 |
| OBP32 | 66.1 | 12.21 | OBP62 | 69.05 | 8.44 |
| OBP33 | 60.66 | 7.15 |  |  |  |

**Figure S1.** OBP gene cluster presents in scaffold NWSH01000044.1 (scaffold length = 319,136 bp). Genes are depicted as triangles: red indicates forward transcription (+) and blue indicates reverse transcription (-).

**Figure S2.** The inferred OR gene tree reconciled with the species tree. Nodes are labeled as D (duplication), S (speciation), or T (horizontal gene transfer). Species are colored as follows: *C. virescens* (pink)*, H. zea* (green)*, H. armigera* (purple)*,* and *B. mori* (black). Duplications involving genes located on the same scaffold and in adjacent positions are highlighted in light yellow.

**Figure S3.** The inferred OBP gene tree reconciled with the species tree. Nodes are labeled as D (duplication), S (speciation), or T (horizontal gene transfer). Species are colored as follows: *C. virescens* (pink)*, H. zea* (green)*, H. armigera* (purple)*,* and *B. mori* (black). Duplications involving genes located on the same scaffold and in adjacent positions are highlighted in light yellow.

**Figure S4.** Identification of conserved motifs in HvirORs via MEME.

**Figure S5.** Identification of conserved motifs in HvirOBPs via MEME.

**Figure S6.** The Shannon entropy distribution of the pocket and non-pocket regions in (A) HvirORs and (B) HvirOBPs.

**Supplemental Data**

HvirORs.fasta: Protein sequences of *C. virescens* ORs

HvOBPs.fasta: Protein sequences of *C. virescens* OBPs

HvirOR_OBP_cDNA.fasta: cDNA sequences of ORs and OBPs in *C. virescens*

OBP_phylogenetic.fa: Protein sequences of OBPs used in the phylogenetic analysis

OR_phylogenetic.fa: Protein sequences of ORs used in the phylogenetic analysis

ModelSelection_OR.txt: Protein substitution model selection analysis of *C. virescens* ORs identified VT+F+R8 as the best-fit model based on BIC scores.

ModelSelection_OBP.txt: Protein substitution model selection analysis of *C. virescens* OBPs identified VT+R5 as the best-fit model based on BIC scores.
